# Glycosylation of a key cubilin Asn residue results in reduced binding to albumin

**DOI:** 10.1016/j.jbc.2022.102371

**Published:** 2022-08-13

**Authors:** Shiv Pratap Singh Yadav, Aiying Yu, Jingfu Zhao, Jasdeep Singh, Saloni Kakkar, Srinivas Chakraborty, Yehia Mechref, Bruce Molitoris, Mark C. Wagner

**Affiliations:** 1 Nephrology Division, Department of Medicine, Indiana University School of Medicine, Indianapolis, Indiana, USA; 2 Department of Chemistry and Biochemistry, Texas Tech University, Lubbock, Texas, USA; 3 Department of Biochemical Engineering and Biotechnology, Indian Institute of Technology Delhi, New Delhi, India; 4 CSIR-Institute of Microbial Technology, Chandigarh, India; 5 BioCAT Beamline APS, Illinois Institute of Technology, Chicago, Illinois, USA; 6 Department of Cellular & Integrative Physiology, Indiana University School of Medicine, Indianapolis, Indiana, USA

**Keywords:** cubilin, albumin, proximal tubule, cell biology, glycosylation, small-angle X-ray scattering (SAXS), kidney, ACN, acetonitrile, BBM, brush border membrane, BS3, bis(sulfosuccinimidyl)suberate, CUB, complement C1r/C1s, Uegf, Bmp1, EGF, epidermal growth factor, IF, intrinsic factor, MALS, multi angle light scattering, MGO, methylglyoxal, MST, microscale thermophoresis, MWF, Munich Wistar Fromter, PT, proximal tubule, RSA, rat serum albumin, SAXS, small-angle X-ray scattering, SEC-MALS, size-exclusion chromatography and multi angle light scattering, SEC-SAXS, Size exclusion chromatography and small angle X-ray scattering, XL-MS, crosslinking mass spectrometry

## Abstract

Kidney disease often manifests with an increase in proteinuria, which can result from both glomerular and/or proximal tubule injury. The proximal tubules are the major site of protein and peptide endocytosis of the glomerular filtrate, and cubilin is the proximal tubule brush border membrane glycoprotein receptor that binds filtered albumin and initiates its processing in proximal tubules. Albumin also undergoes multiple modifications depending upon the physiologic state. We previously documented that carbamylated albumin had reduced cubilin binding, but the effects of cubilin modifications on binding albumin remain unclear. Here, we investigate the cubilin-albumin binding interaction to define the impact of cubilin glycosylation and map the key glycosylation sites while also targeting specific changes in a rat model of proteinuria. We identified a key Asn residue, N1285, that when glycosylated reduced albumin binding. In addition, we found a pH-induced conformation change may contribute to ligand release. To further define the albumin-cubilin binding site, we determined the solution structure of cubilin’s albumin-binding domain, CUB7,8, using small-angle X-ray scattering and molecular modeling. We combined this information with mass spectrometry crosslinking experiments of CUB7,8 and albumin that provides a model of the key amino acids required for cubilin-albumin binding. Together, our data supports an important role for glycosylation in regulating the cubilin interaction with albumin, which is altered in proteinuria and provides new insight into the binding interface necessary for the cubilin–albumin interaction.

Albuminuria is an established risk factor for the progression of chronic kidney disease to end-stage kidney disease, cardiovascular disease, and mortality (1, 2, 3, 4). Studies have documented both glomerular and proximal tubule (PT) alterations that can lead to albuminuria (5). Glomerular albumin filtration is accepted though levels of albumin filtration remain unresolved (6). Filtered albumin will arrive at the PT with associated ligands and various modifications depending upon physiologic state. Thus, the response of the PT will be dictated by whether albumin is endocytosed by clathrin-dependent or clathrin-independent endocytosis, the resultant signaling and trafficking events. The large, ∼400 kDa, PT endocytic receptor cubilin (CUBN) is a brush border membrane (BBM) glycoprotein that binds albumin and interacts with the transmembrane protein amnionless (AMN) and the multiligand PT BBM endocytic receptor megalin (>500 kDa) to facilitate albumin endocytosis (7, 8). Albumin is then directed to the lysosomal degradation pathway or *via* a receptor handoff to FcRn, the transcytotic salvage pathway (9, 10). The mechanism of this cubilin to FcRn handoff is thought to involve both pH and Ca changes. Alterations in both cubilin and FcRn have led to increases in albuminuria and altered plasma albumin levels (5, 11, 12, 13, 14). The binding mechanism and regulation of cubilin–albumin and cubilin–megalin interactions are not well understood and require more investigation. Adding to the complexity of these interactions is the role of glycosylation since both cubilin and megalin have ∼40 predicted glycosylation sites (15, 16, 17).

CUBN was first defined as the gastric intrinsic factor (IF)-cobalamin (Cbl,vitamin B_12_) receptor, a critical function given that biosynthesis of B_12_ is restricted to prokaryotes (18, 19). Additional studies led to the purification of cubilin, identification of its multiple domains, Figure 1, and its calcium-dependent interaction with megalin (20, 21, 22). Cubilin contains a coiled-coil N-terminal region, eight epidermal growth factor-like (EGF-like) domains followed by 27 CUB (complement C1r/C1s, Uegf, Bmp1) domains (18, 21, 22). Subsequently, it was found to have a novel interaction with the type-1 transmembrane protein AMN to form the single or double stem (∼2.8 MDa) Cubam complex (23). Increased understanding of the Cubam complex comes from genetic analyses of Imerslund-Grasbeckj syndrome or juvenile megaloblastic anemia which are caused by mutations in either AMN or CUBN (14, 24, 25, 26). These studies established that the AMN and CUBN interaction was essential for cell surface targeting of the Cubam complex and identified four cubilin N-glycosylation sites necessary for surface expression (27, 28). Protein expression and analyses of specific cubilin domains showed CUB5–8 to contain the IF-Cbl and albumin-binding sites. When IF-Cbl is bound to CUB5–8, albumin binding is reduced while IF-Cbl binding was not altered by albumin binding (21). These series of studies also identified Alpha-2-macroglobulin receptor-associated protein (Lrpap1 or RAP), a molecular chaperone (29), binding domain (CUB13&14), and found three regions (N-terminus +EGF +CUB1&2, CUB12–17 domain, and CUB22–27 domain) of cubilin that bound to megalin in a Ca^2+^-dependent manner (20, 22).

**Figure 1:**
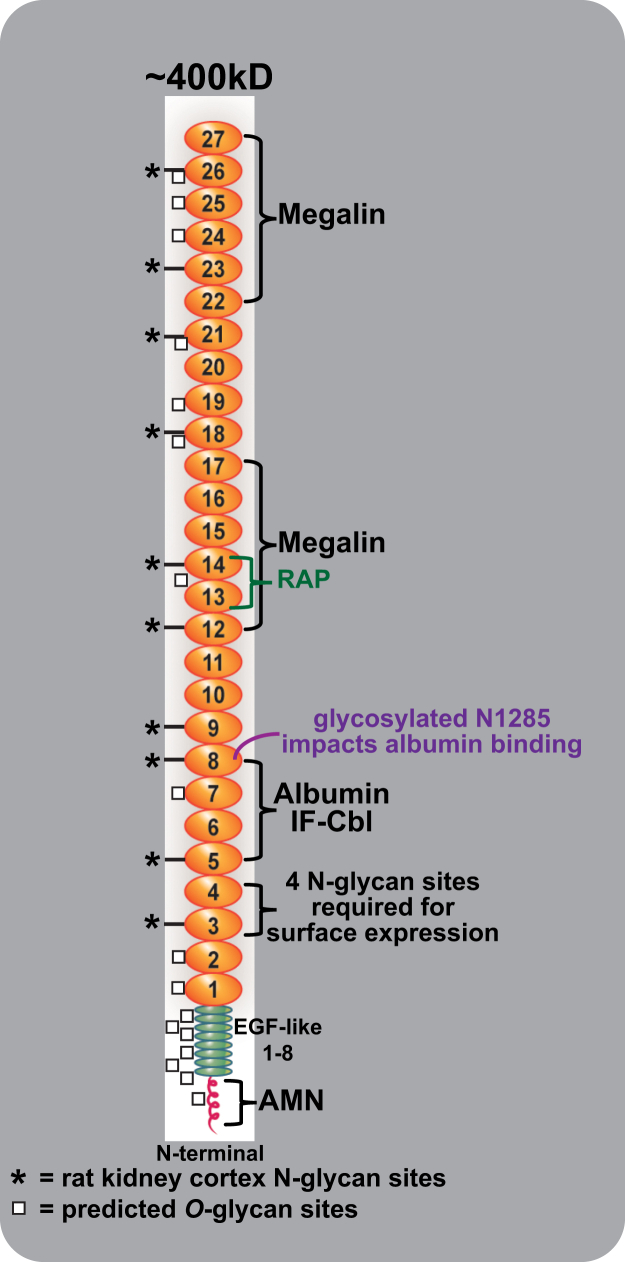
**Cubilin domains, binding partners, and glycosylation sites.** Cubilin is a peripheral membrane protein containing an N-terminal stretch of 110 amino acids, eight EGF-type repeats, and 27 CUB domains. Protein expression and analyses of specific cubilin domains showed CUB5–8 to contain the IF-B12 and albumin-binding sites, identified Alpha-2-macroglobulin receptor associated protein (RAP) binding domain (CUB13&14), and found three regions (N-terminus +EGF +CUB1&2, CUB12–17 domain and CUB22–27 domain) of cubilin that bound to megalin in a Ca^2+^-dependent manner. It also has a novel interaction with the type-1 transmembrane protein amnionless (AMN), which forms the Cubam complex. Studies from genetic analyses of Imerslund-Grasbeckj syndrome or juvenile megaloblastic anemia (IGS or MGA1) which are caused by mutations in either AMN or CUBN identified four cubilin *N*-glycosylation sites necessary for surface expression. The squares identify predicted *O*-glycan sites for cubilin using the NetOglyc 4.0 Server, while the asterisk identify the location of the 10 N-glycosylation sites we identified in cubilin isolated from rat kidney cortex. CUB, complement C1r/C1s, Uegf, Bmp1; EGF, epidermal growth factor; IF, intrinsic factor.

Given the importance of cubilin in PT albumin endocytosis a more complete understanding of this interaction, its regulation and how it is impacted in disease states is warranted. We previously documented carbamylated albumin had reduced binding to CUB7,8 (30) and now report similar reduction in glycated albumin. Reduced binding of albumin to cubilin could lead directly to albuminuria. We have also shown significant changes in the glycome of the PT BBM with disease (31, 32) which is known to impact cell signaling, interactions, and ligand binding. Our investigation of glycosylation of the CUB7,8 domain in both *in vitro* and *in vivo* studies supports the posttranslational modification can have an important regulatory role in cubilin–albumin interactions and thus may directly impact albumin processing. Our studies have quantified their binding, evaluated their structural association, and explored glycosylations impact on this interaction. We chose to focus on the CUB7,8 domain as our *in vitro* microscale thermophoresis (MST) binding assay, Figure 3, was consistent with that shown previously using surface plasmon resonance with albumin and purified rat cubilin immobilized to a BIAcore sensor chip (33). The biophysical analyses undertaken identified multiple cubilin glycosylation sites that impact albumin binding, identified amino acids in this binding interface using crosslinking mass spectrometry (XL-MS) combined with small-angle X-ray scattering (SAXS), and molecular modeling, allowed a solution structure of the cubilin-albumin binding domain to be proposed. Finally, we document *in vivo* glycan changes to the specific CUB7,8 glycan site, which is the most impacting albumin binding in an animal model of proteinuria.

## Results

### Investigation of cubilin N-glycosylation and impact on albumin interaction

**Figure 2:**
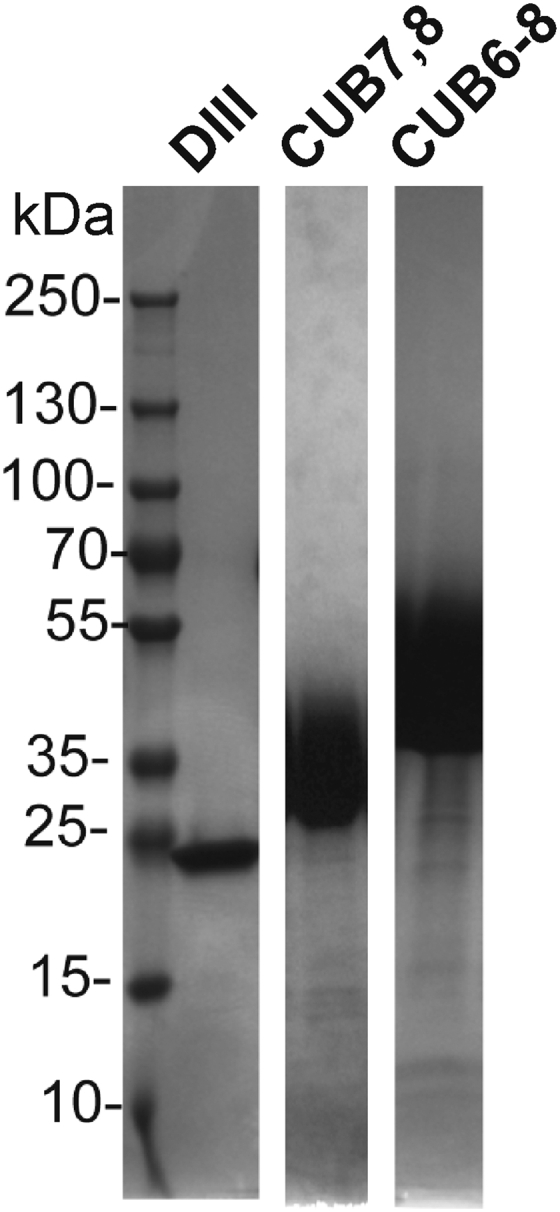
**Purified 293F-expressed proteins.** The purified proteins were separated on SDS-PAGE gels and stained with Coomassie Blue. The migration of the nine prestained molecular weight standards are shown on the *left*. DIII (Albumin domain III) migrates as expected for a 22.7 kDa protein. CUB7,8 (Cubilin 7,8 domain predicted molecular weight of 26.2 kDa) and CUB6–8 (Cubilin 6–8 domain predicted molecular weight of 39.3 kDa) migrate as diffuse bands above their predicted MW consistent with being glycosylated. CUB, complement C1r/C1s, Uegf, Bmp1.

Cubilin was first defined by the Moestrup laboratory as a megalin-binding protein that was glycosylated as shown by a reduction in SDS-PAGE migration following PNGaseF treatment (18). The Seetharam laboratory used an *in vitro* translation system and ligand affinity chromatography to identify the binding region for gastric IF cobalamin (vitamin B_12_; Cbl), IF-Cbl, and albumin (21). Only the proteins containing cubilin domains 7 & 8 bound both IF-Cbl and albumin. To further investigate the albumin-binding region, the CUB7,8 & CUB6–8 domains were expressed in 293F cells, see Figure 2.

**Figure 3:**
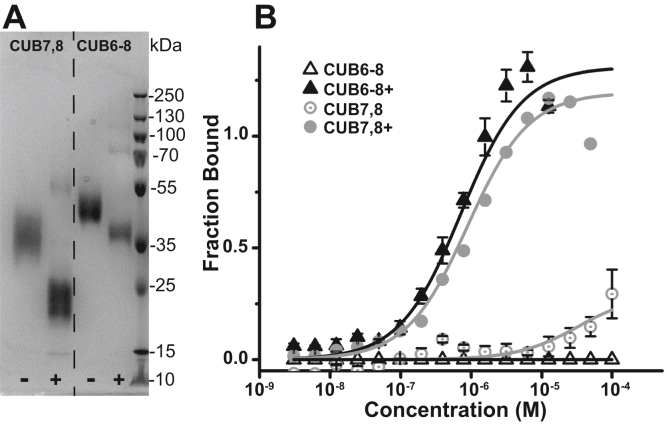
**Cubilin 7,8 & 6–8 bind albumin only after PNGaseF treatment.***A*, purified cubilin domains were deglycosylated using PNGaseF, both CUB7,8 & CUB6–8 are shown before PNGaseF treatment (−) and after PNGaseF treatment (+). Note the migration of both cubilin domains is consistent with the removal of N-glycans by PNGaseF resulting in the predicted MW for CUB7,8 of 22.7 kDa and 39.3 kDa for CUB6–8. *B*, Cubilin binding to albumin was determined using Microscale thermophoresis, MST. Only when cubilin was treated with PNGaseF + (○-CUB7,8 & ▲-CUB6–8) did binding occur. In the absence of PNGaseF treatment (○-CUB7,8 & Δ-CUB6–8), binding was very low or undetected. CUB, complement C1r/C1s, Uegf, Bmp1; MST, MicroScale Thermophoresis.

Given the established importance of membrane receptor glycosylation in trafficking, ligand binding, and regulation, we first asked whether the expressed cubilin domains were glycosylated and then whether albumin binding was impacted by this modification. Figure 3*A* shows both CUB7,8 and 6–8 bands −/+ PNGaseF treatment. Note the increased SDS-PAGE migration of both CUB7,8 & CUB6–8, consistent with our predicted molecular weight based on each sequence, see Fig. S1 and the MW determined by MALDI-MS, Fig. S2. Figure 3*B* shows the MST-binding results documenting that albumin binding only occurs after PNGaseF treatment for both domains (Kd for CUB7,8 = 0.5–1.2 μM and for CUB6–8 = 0.5–1.0 μM, glycosylated domains had Kd > 100 μM or not detected). Note, this Kd is consistent with the reported Kd of 0.63 μM for purified (by IF-B_12_ affinity column) rat cubilin binding to rat albumin using BIAcore sensor chips (33).

Determining how cubilin glycosylation can impact albumin binding requires defining the glycans and their location. As a first step, CUB7,8 before and after PNGaseF treatment was analyzed using MS/MS methods. Table 1 lists the five potential glycosylation site sequences and the number of glycan structures detected. Note, all the CUB7,8 asparagine glycosylation sites are conserved in bovine (UniProtKB-F1MKV7), human (UniProtKB-O60494), mouse (UniProtKB-Q9JLB4), and rat (UniProtKB-O70244) cubilin. Table S2 lists each glycan structure detected at each site. Also, various unknown modifications were detected for Cubilin 8 site 1 and four *O*-glycopeptides were identified in the cubilin 7 domain. These results are presented in Fig. S3 and their possible significance requires further investigation. To evaluate whether albumin binding could be achieved by preventing glycosylation at Cubilin 8 site 1, the Asn 1285 was changed to either a Pro or Gln. Neither of these changes resulted in albumin binding without PNGaseF glycosidase treatment, Fig. S4 (binding data shown for CUB7,8-N1285P but did result in increased glycosylation at four locations in CUB7,8 as shown in Table 2). Testing the effect of amino acid changes at multiple CUB7,8 Asn glycosylation sites to further reduce glycosylation were unsuccessful as protein was not expressed in the 293F cells, possibly due to incorrect folding and instability (34). We have begun an analysis of other glycosidases to determine if incomplete trimming can result in albumin binding.

**Table 1:** CUB7,8 N-glycosylation sites

N-glycosylation site sequence	Associated glycan structure (refer to Table S1 for details)
CGG**N**LTTPTGVL (Cubilin 7), N1168	Glycopeptides were not confirmed with MS^2^, but deglycosylated peptides were identified in PNGaseF samples at both MS^1^ and MS^2^ levels.
CDNVVIV**N**K (Cubilin 8 site 1), N1285	36 structures (High mannose, fucosylated and sialylated)
C**N**W (Cubilin 8 site 2), N1307	2 structures (High mannose, fucosylated and sialylated)
TIQATTGNTV**N**Y (Cubilin 8 site 3), N1319	9 structures (High mannose and complex)
M**N**CSTDY (Cubilin 8 site 4), N1332	Not detected

The **N** in bold signifies the glycosylated asparagine residue.

**Table 2:** Cubilin 8 site 1 (CDNVVIVNK). 1285N to P or 1285N to Q change

N-Glycosylation site sequence	N to P glycans	N to Q glycans
CGG**N**LTTPTGVL (Cubilin 7),N1168	51	13
DK**N**QRCNW (Cubilin 8), N1303	29	18
TIQATTGNTV**N**Y (Cubilin 8 site 3), N1319	59	29
M**N**CSTDYVELY (Cubilin 8 site 4), N1332	0	0

The **N** in bold signifies the glycosylated asparagine residue.

### Binding of cubilin to modified albumins

**Figure 4:**
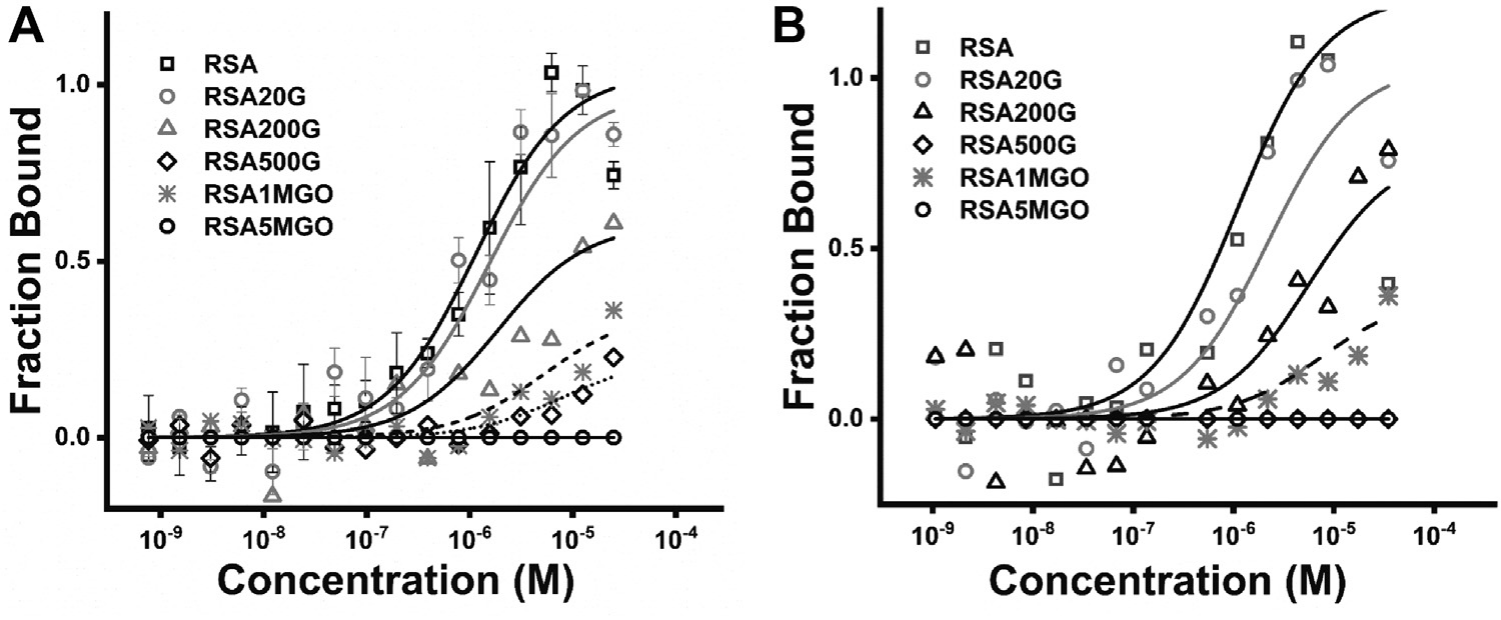
**Albumin glycation decreases cubilin binding.***A*, PNGaseF-deglycosylated CUB7,8 showed a concentration-dependent decrease in albumin binding as glycation was increased. Binding affinity (Kd mM) was 0.5 to 1, 2.5, 25.6, >100 or ND, 35, and >100 or ND for RSA, RSA 20 mM glucose (RSA20G), RSA 200 mM glucose (RSA200G), RSA 500 mM glucose (RSA500G), RSA 1 mM methylglyoxal (RSA1MGO), and RSA 5 mM methylglyoxal (RSA5MGO), respectively. *B*, PNGaseF-deglycosylated CUB6–8 showed a similar concentration-dependent decrease in albumin binding as glycation was increased. Binding affinity (kD mM) was 0.5 to 1, 2, 9.4, >100 or ND, 28, and >100 or ND for RSA, RSA 20 mM glucose (RSA20G), RSA 200 mM glucose (RSA200G), RSA 500 mM glucose (RSA500G), RSA 1 mM methylglyoxal (RSA1MGO), and RSA 5 mM methylglyoxal (RSA5MGO), respectively. CUB, complement C1r/C1s, Uegf, Bmp1.

We previously reported that cubilin and FcRn had reduced binding to carbamylated albumin (30) and that glycated albumin binding to FcRn (35) was also reduced. To determine if glycated albumin had altered binding to cubilin, binding of PNGaseF deglycosylated CUB7,8 (Fig. 4*A*) and CUB6–8 (Fig. 4*B*) domains were performed. Note, the concentration-dependent binding inhibition for both glucose (G) and methylglyoxal (MGO) modified albumin and increased inhibition for the MGO-treated albumin, similar to what we reported for FcRn binding (35). We also confirmed that both CUB7,8 and CUB6–8 had normal binding to the albumin FcRn mutant, H464A. In addition, both CUB7,8 and CUB6–8 bound to albumin DIII H464A, further indicating the binding interface lies somewhere in DIII domain, Fig. S6.

### Investigation of albumin species-cubilin binding differences and impact of pH and Ca^2+^

**Figure 5:**
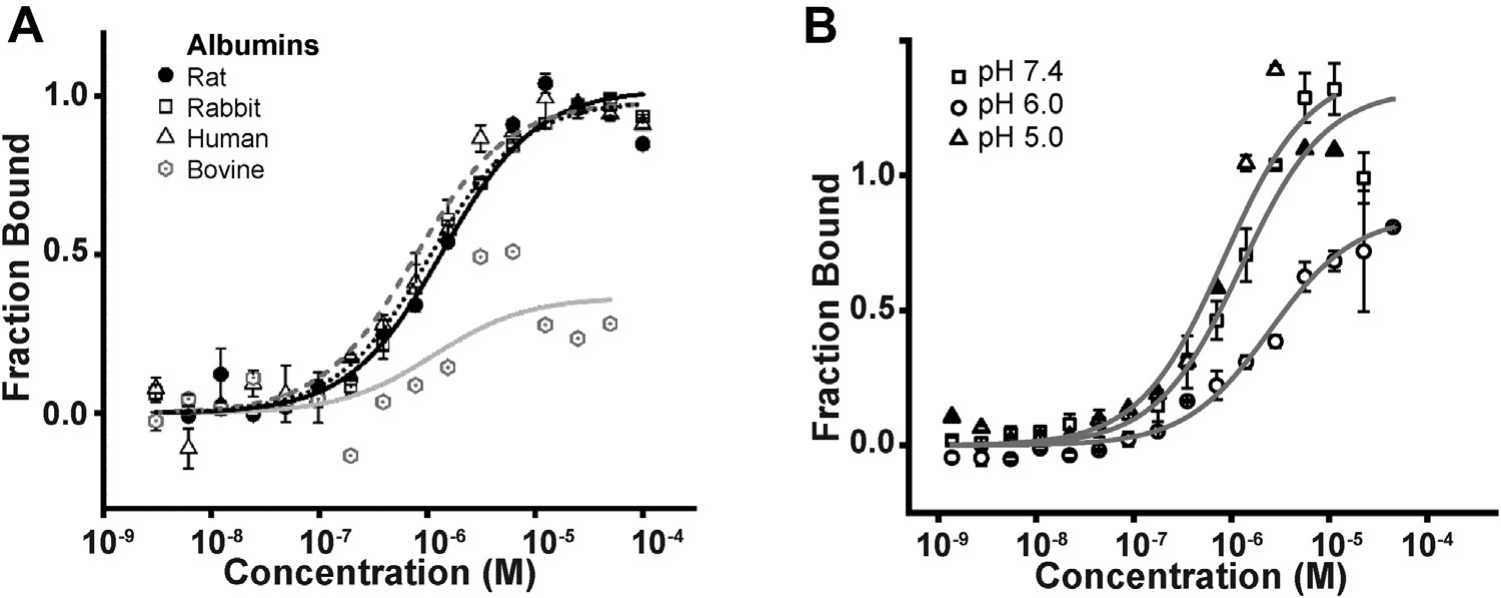
**Species differences in cubilin-albumin binding and the impact of pH.***A*, PNGaseF-deglycosylated CUB7,8 bound (Kd in mM) similarly to rat (0.5–1.0), rabbit (0.9–1.4), and human (0.95–1.5) albumin but had reduced binding to bovine albumin (>100). *B*, PNGaseF-deglycosylated CUB7,8 had a small decrease in albumin binding (Kd in mM) as pH decreased. Note, the largest decrease in binding was observed at pH 6.0 (1.7) *versus* pH 7.4 (0.5–1.0) and pH 5.0 (1.4). CUB, complement C1r/C1s, Uegf, Bmp1.

**Figure 6:**
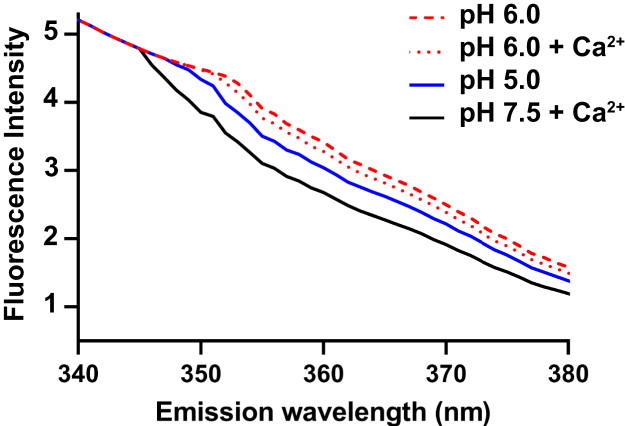
**Cubilin Tryptophan fluorescence.** Intrinsic tryptophan fluorescence changes in CUB6–8 with pH and calcium. A fluorescence emission scan (340–400 nm), 1 nm steps, was measured with excitation of 280 nm (280-10) and a dichroic long pass filter of 310 nm. Fluorescence intensity is expressed in arbitrary units and measurements were made using the Clariostar plate reader. Note the largest difference was between CUB6–8 at pH 6.0 (*red dashed line*) and CUB6–8 pH 7.5 + calcium (*solid black line*). CUB, complement C1r/C1s, Uegf, Bmp1.

To investigate whether rat CUB7,8 bound differently to albumin from other species, rat, rabbit, human, and bovine albumin were compared. Similar binding affinity (Kd ∼ 1 μM) was found for rat (0.5–1.2 μM), rabbit (0.9–1.4 μM), & human (0.95–1.5 μM) albumin, while bovine albumin binding was very weak Kd >100 μM, Figure 5*A*. Albumin species binding differences to its transcytotic receptor FcRn have also been reported (35, 36, 37, 38). As mentioned previously, a direct role for the cubilin/megalin multiligand receptor directly participating in a portion of PT albumin endocytosis is well established. What is less understood is how endocytosed albumin is transferred to its only known transcytotic receptor, FcRn (12). This handoff could occur if cubilin releases albumin at low pH since FcRn only binds albumin at pH <7.4 (39). Figure 5*B* supports this hypothesis by showing a slight decrease in CUB7,8 albumin binding at pH 6.0 and 5.0. Endosome pH ranges from >pH 6.0 in early endosomes to around pH 5.0 by late endosomes with lysosomes having a pH between 4.6 and 5.0 (40). In addition, fluctuation of other ions including Cl^−^, Na^+^, K^+^, and Ca^2+^ also occur during endosome transport (41). Our pH binding results suggest albumin releases from cubilin prior to reaching late endosomes. Two early studies supported a role for Ca^2+^ in albumin cubilin binding (21, 33). Since our MST attempts to evaluate Ca^2+^ impact on CUB7,8 binding were inconclusive, we addressed a role for both pH and Ca^2+^ in ligand binding by measuring tryptophan fluorescence differences to determine whether any conformation change in CUB7,8 or CUB6–8 proteins could be detected (42, 43, 44). Note, CUB6–8 contains multiple Ca^2+^-binding sites, disulfide bonds, and seven Trp residues. In addition, disulfides are necessary for binding of both albumin and IF-Cbl to cubilin (21). While both proteins showed similar shifts with pH and calcium, the magnitude of the change was greatest in CUB6–8 when comparing pH 7.5 + Ca^2+^ to pH 6.0 − Ca^2+^, Figure 6. This result in combination with our pH result is consistent with a change, that is, albumin release from cubilin taking place in an endosome with pH 6.0 and reduced Ca2+. A pH & Ca^2+^-induced conformation change in cubilin could reduce albumin binding thus promoting FcRn binding of albumin and enabling ligand sorting and eventual transcytosis. Note, while we did not observe an impact of Ca^2+^ on CUB7,8 and albumin binding, this may be due to the small cubilin domain used for monitoring binding and a pH & Ca^2+^ conformation change. In the large native protein, a change comparable to what has been observed for other recycling receptors such as the low-density lipoprotein may result in altered ligand binding (42, 45). Future innovative intravital approaches may reveal the specific pH and Ca^2+^ conditions for the dynamic handoff of albumin from cubilin to FcRn.

### Structural studies aimed at understanding cubilin-albumin binding interaction and site

#### Shape/structural and biophysical parameters using SEC-MALS-SAXS

**Figure 7:**
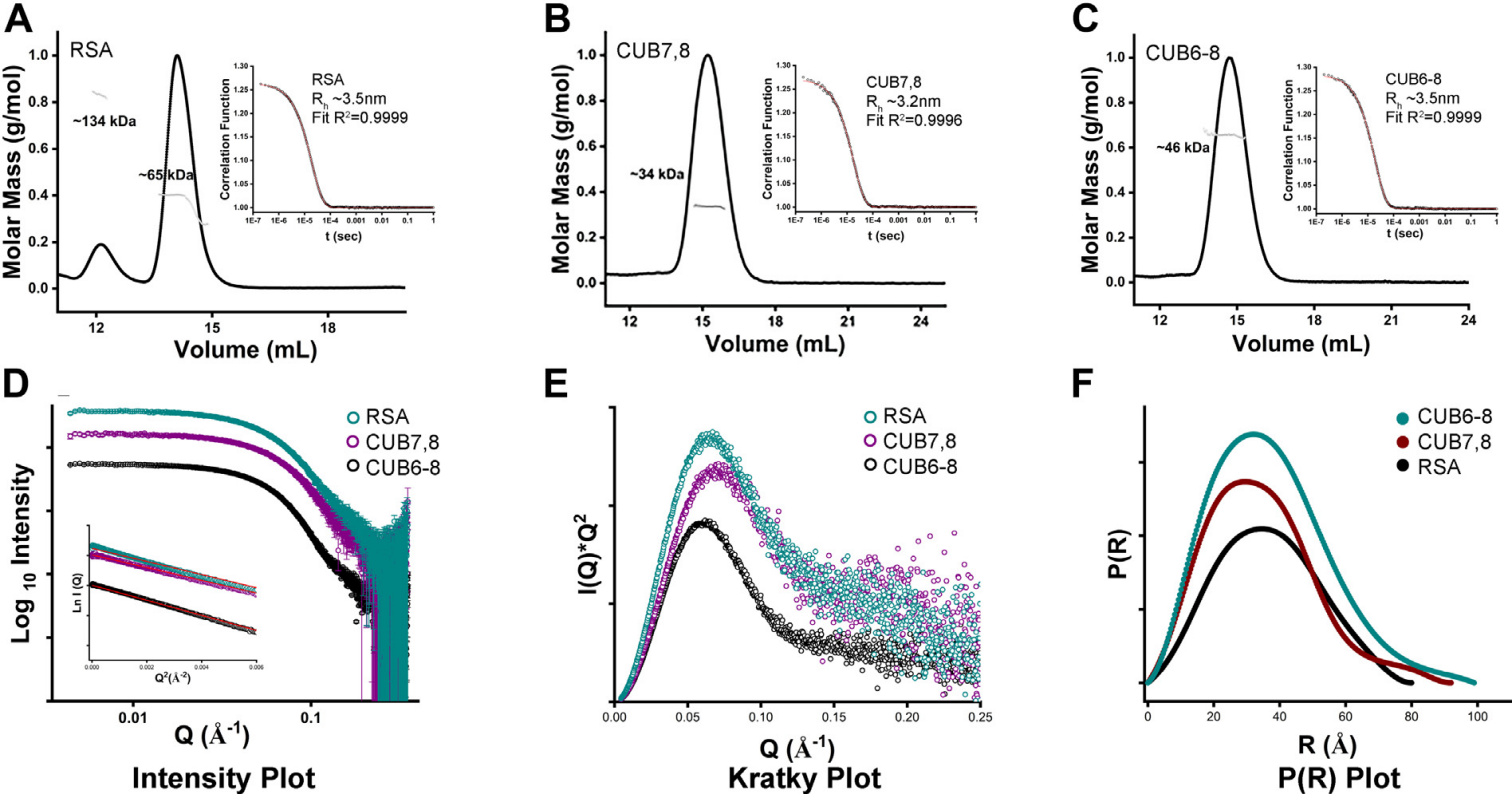
**SEC-MALS-SAXS -RSA, CUB7,8 6–8.** Multi angle light scattering (MALS) plots showing the molecular mass peak profiles for RSA (minor peak at ∼134 kDa representing a dimer and major peak at ∼65 kDA for the monomer, *A*), CUB7,8 (peak at ∼34 kDa, *B*), and CUB6–8 (peak at ∼46 kDa, *C*) as calculated by protein concentration and X-ray scattering intensity. Inset shows the Hydrodynamic radii, R_H_, of each scattering species calculated using Autocorrelation function (ACF). The SAXS scattering intensity profiles of RSA, CUB7,8, and CUB6–8 are plotted as Log_10_ Intensity (log_10_ I(Q)) *versus* Scattering vector (Q) in (*D*) with their respective linearity in the Guinier region presented in the inset showing monodispersity for all proteins. The Kratky plot is presented in (*E*) showing a bell-shaped curve consistent with each protein having a globular scattering profile. Finally, the plot in (*F*) presents pairwise distribution, P(R) histogram of small and large scattering vectors, showing the extent of maximum linear dimensions (D_max_) for each protein. See Table 3 for R_G_ D_max_ values. Note, both cubilin proteins were purified from 293F cell supernatant as described in Experimental procedures and not treated with any glycosidase prior to this analysis. CUB, complement C1r/C1s, Uegf, Bmp1; SEC-MALS, size-exclusion chromatography and multi angle light scattering.

**Figure 8:**
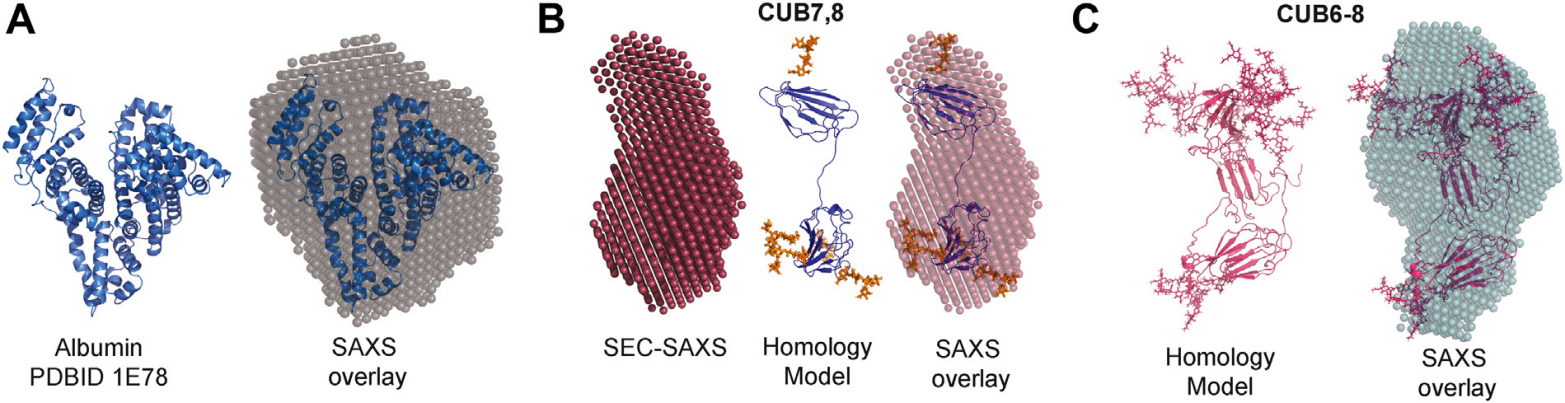
**SEC-SAXS derived SAXS envelopes.***A*, albumin monomer scattering intensity profile was overlaid on crystal structure PDB ID 1E78, SAXS envelopes were generated using uniform density models (DAMMIF) from X-ray scattering profiles, and data were collected at BIOCAT beamline., ANL. *B*, CUB7,8 uniform density experimental SAXS envelope (*magenta*) was overlaid on homology model of glycosylated CUB7,8 (*blue cartoon*) by I-TASSER and Glyprot, taking PDB ID 3KQ4 as reference in the middle and then overlaid on SAXS envelope. *C*, glycosylated CUB6–8 homology model obtained (*pink cartoon*) by I-TASSER and Glyprot (PDB ID 3KQ4 as reference) was superimposed on SEC-SAXS generated model. CUB, complement C1r/C1s, Uegf, Bmp1; SEC-SAXS, size-exclusion chromatography and small angle X-ray scattering.

Multi angle light scattering (MALS) incorporated with size-exclusion chromatography showed two clear peaks of rat serum albumin (RSA) as monomer and dimer (∼65 kDa, ∼134 kDa) Figure 7*A*, which is a positive control as well as an interacting partner for cubilin. Smaller CUB7,8 (glycosylated) domain protein showed predominantly monomer peak and hydrodynamic radii of 3.2 nm and ∼34 kDa molecular weight, Figure 7*B*, while three domain CUB6–8 (glycosylated) protein also showed a monomer state in solution with a hydrodynamic radius of 3.5 nm and molecular weight of ∼46 kDa, Figure 7*C*. These values agree with the estimated molecular weight of Cubilin domains as calculated by MALDI-MS (Fig. S2) and estimated by homology modeling. Unexpectedly, CUB7,8N1285P mutant, which is glycosylated less than CUB7,8, showed two peaks monomer at 36 kDa and a likely dimer at ∼ 71 kDa, data not shown. Size-exclusion chromatography and multi angle light scattering (SEC-MALS) was followed by SAXS, which was used to compute the shape parameters of Cubilin domains, In Figure 7*D*, intensity profiles obtained from SAXS data are plotted Log_10_ IQ *versus* Q, which indicates no aggregation and monodispersity for RSA (control), CUB7,8 and CUB6–8 which assures the quality of scattering species. In the inset of Figure 7*D*, Guinier region is plotted as natural log of Intensity as a function of scattering vector (q^2^), resulting in linear slope which is proportional to the square of the radius of gyration (Rg). After quality assurance of the scattering species, Kratky analysis was performed to reveal the nature of proteins in solution, RSA (control) which shows an expected globular behavior confirmed by bell shaped curve (Fig. 7*E*), while CUB7,8 and CUB6–8 shows a slight ascending bell-shaped curve, pointing toward the disordered linker regions in Cubilin domains. Figure 7*F* shows the maximum linear dimensions as computed by pairwise distribution function P(R) and are plotted as P(R) *versus* R, whose values are mentioned in Table 3. Shape parameters of RSA were calculated as shown in our previous studies, Table 3 (30, 35). Cubilin domains showed an elliptical and elongated shape of Rg and Rc of 2.62 nM and 2.86 nm, while D_max_ of 9.1 nm and 9.9 nm. The molecular weight calculated by SAXS parameters and DATMOW, Vc were 34.15 and 49.9 kDa and are remarkably close to molecular weight estimation by SEC-MALS and modeling. Uniform density modeling, taking P1 symmetry into account, showed a predominant monomer for both cubilin domains. Figure 8*A* shows the crystal structure of RSA (control) perfectly fitted into SAXS envelope. In the absence of X-ray crystallography data for Cubilin, homology models of CUB7,8 and CUB6–8 were created for glycosylated CUB7,8 (Fig. 8*B*) and CUB6–8 proteins (Fig. 8*C*).

**Table 3:** Structural parameters of RSA, Cub7,8, and Cub6-8 as deduced from Guinier analysis and indirect Fourier transformation of small-angle X-ray scattering data

Protein	Guinier analyses				Indirect Fourier transform			
	mg/ml	R_G_ (Å)	R_C_ (Å)	L (Å)	R_G_ (Å)	D_max_ (Å)	Datmow (Da)	Primary state
RSA	∼3	27.8	17.0	77.5	27.9	81	63,102	Monomer
Cub7,8	∼4	26.2	14.0	77.6	26.6	91.1	34,157	Monomer
Cub6–8	∼4	28.6	15.6	77.4	28.9	99	49,913	Monomer

D_max_, maximum linear dimension; *L*, persistant length; *R*_c_, radius of cross-section; *R*_g_, radius of gyration.

**Figure 9:**
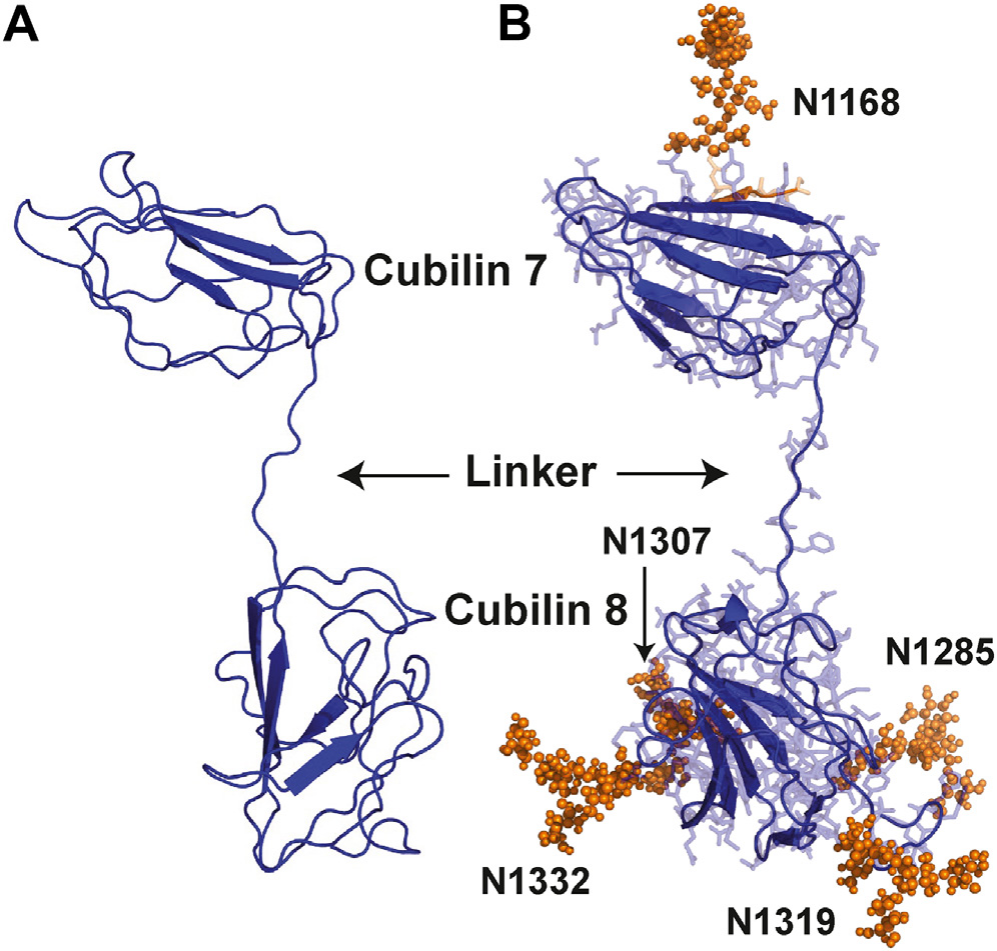
**Models of nonglycosylated and glycosylated CUB7,8.***A*, homology model of CUB7,8 (PDB ID 3KQ4). *B*, homology model glycosylated CUB7,8 (gCUB7,8), glycosylation sites predicted by Glyprot server to build glycosylated model and verified by MS data (N1168, N1285, N1307, N1319, N1332). *Blue color* cartoon represents CUB7,8 homology model derived from CUB5–8, PDB ID 3KQ4. CUB, complement C1r/C1s, Uegf, Bmp1.

Phyre2, a suite of tools, was used to predict and analyze protein structures of CUB7,8 and CUB6–8 in extensive mode followed by further verification using I-TASSER (46, 47). This suite uses advanced remote homology detection and loop modeling to build 3D models and *Ab initio* modeling for modeling of loops\missing regions along with backbone and side chain addition using Poing. The best similarity was found with PDB ID 3KQ4 with 59% identity and 100% confidence for CUB6–8, while for CUB7,8, it was 62% identical and a confidence of 100%. Theoretical length (L) was very similar for the proteins CUB7,8 (84 Å) and CUB6–8 (86 Å) which likely reflects the significance of the extended linker in CUB7,8 protein and the N1285 residue, located in the linker region, which is heavily glycosylated Figure 9, see Tables 1 and S2. This site appears to play a pivotal role in maintaining a concave shape of the protein similar to CUB6–8 protein. Glyprot server was used to link glycosylations on CUB7,8 and CUB6–8 as observed in Mass spectrometry and Glyprot predictions (N1168, N1285, N1307, N1319, N1332). These glycosylated models of CUB7,8 and CUB6–8 domain were well fitted into SAXS envelopes (Fig. 8, *B* and *C*) created by DAMMIF function of ATSAS suite with permissible penalties. Size exclusion chromatography and small angle X-ray scattering (SEC-SAXS) data for CUB7,8N1285P mutant showed oligomeric nature in SAXS data, previously observed in SEC-MALS data as well. The shape/structural parameters for CUB7,8N1285P were calculated by considering oligomeric nature. The behavior of CUB7,8N1285P may be due to the deletion of the asparagine, which was glycosylated, see Table 1. Lack of glycosylation at N1285 prevented the stearic hindrance at the linker which connects two domains, which might be the reason for association or oligomerization.

### XL-MS and putative-binding interface

**Figure 10:**
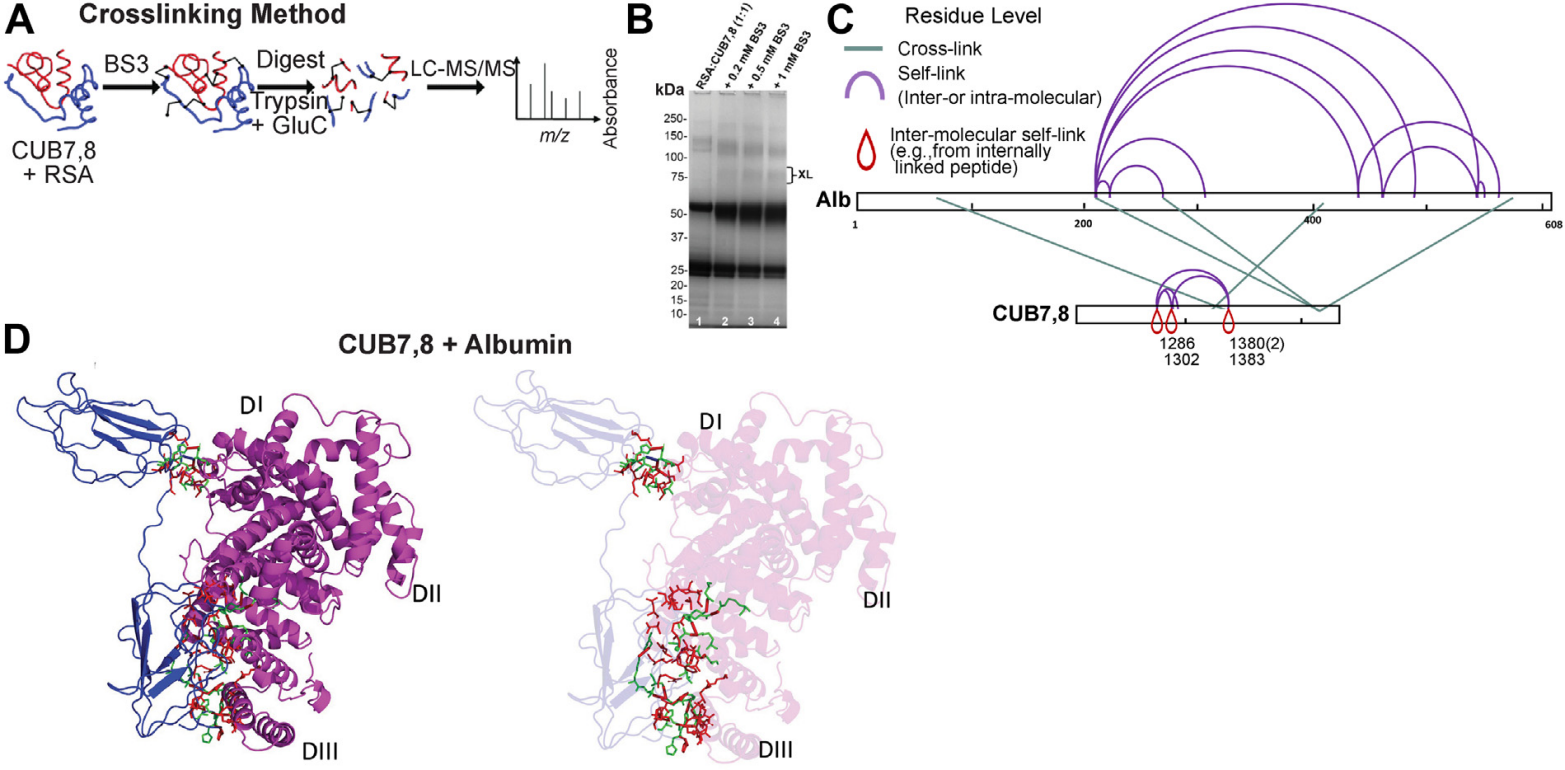
**XL-MS crosslinking methodology and protein–protein docking.***A*, overview of the BS3 Crosslinking steps to investigate interacting CUB7,8 and albumin peptides. *B*, Coomassie blue–stained SDS-PAGE gel showing the appearance of albumin-CUB7,8 proteins in the presence of BS3, XL. Note the increase in intensity of bands with increasing concentration of BS3. *C*, crosslinking map of mass spectrometry–validated BS3 crosslinked residues is shown schematically on albumin and CUB 7,8 proteins. These experimental XL-MS crosslinks were used in computational docking to predict the binding interface. *D*, docked model of CUB7,8 and albumin (based upon XL-MS residual network and docking; *left panel*). Interacting amino acids are highlighted in both CUB7,8 and albumin to identify the docking sites more easily (*right panel*). CUB 7 interacts with DI domain of serum albumin, while most of the interacting residues are present in CUB 8 domain which interacts with DIII domain of albumin. BS3, bis(sulfosuccinimidyl)suberate; CUB, complement C1r/C1s, Uegf, Bmp1; XL-MS, crosslinking mass spectrometry.

The His tagged CUB7,8 domain and albumin were mixed in 1:1 and 1:2 M ratio, and the mixture was incubated for 10 min. Protein complexes were then loaded onto a Ni-NTA column and free proteins were further washed in wash buffer. The eluted complex was incubated for 15 min, room temperature with 1 mM bis(sulfosuccinimidyl)suberate (BS3), a homobifunctional NHS ester, and a water-soluble cross-linker. The bands, at an expected 1:1 complex, were removed and sent for Mass spectrometry analysis. Figure 10*A* shows an overview of the method and Figure 10*B* is the SDS-PAGE gel showing the crosslinked region, XL, used for analysis. After digestion with GluC+Trypsin, crosslinked peptides were subjected to mass spectrometry data collection and analysis. XL-MS showed a majority of cubilin self-association peptides but did reveal five cubilin-albumin crosslinked peptides, Figure 10*C* and Table 4. Complete Spectra of the five cubilin-albumin cross-linked peptides generated using pLable is shown in Fig. S5. Sequence search analysis of inter crosslinked peptides showed primarily albumin’s DIII domain and cubilin eighth domain takes part in binding of albumin-CUB7,8 complex, while few peptides originated from CUB7 domain and DI domain were also observed which points toward nominal binding with these domains, Figure 10*D*. In addition, albumin domain III alone binds to cubilin with similar affinity to albumin as shown in Fig. S6. Also, CUB7,8 self-associated crosslinked peptides were found, as expected if these domains contribute to cubilin self-association as shown previously (8, 48).

**Table 4:** XL-MS analysis of cross-linked CUB7,8 and albumin

Band 1 treated with GluC + trypsin crosslinked peptides					
Peptide order	Peptide	Peptide mass	Modifications	Proteins	Protein type
1	INKECCHGDLLE(3)-KGFK(1)	2104.0201	Carbamidomethyl[C](5); Carbamidomethyl[C](6)	sp|P02770|ALBU_RAT(267)-sp|O70244|CUBN_RAT(1380)/	Inter-Protein
2	NVVIVNKTSGILE(7)-PKNLVK(2)	2221.3165	null	sp|O70244|CUBN_RAT(1286)-sp|P02770|ALBU_RAT(409)/	Inter-Protein
3	SINYPNPYDKNQR(10)FAKTCVADENAE(3)	3100.4315	Carbamidomethyl[C](21)	sp|O70244|CUBN_RAT(1302)-sp|P02770|ALBU_RAT(75)/	Inter-Protein


Band 2 treated with GluC + trypsin crosslinked peptides					
Peptide order	Peptide	Peptide mass	Modifications	Proteins	Protein type
1	GFKMQWFTHGHHHH(3)-LDAVKE(5)	2614.241	Oxidation[M](4)	sp|O70244|CUBN_RAT(1383)-sp|P02770|ALBU_RAT(210)/	Inter-Protein
2	GINSGEKGFK(7)-DQLKTVMGD(4)	2196.085	Oxidation[M](20)	sp|O70244|CUBN_RAT(1380)-sp|P02770|ALBU_RAT(569)/	Inter-Protein

### Docking results

**Figure 11:**
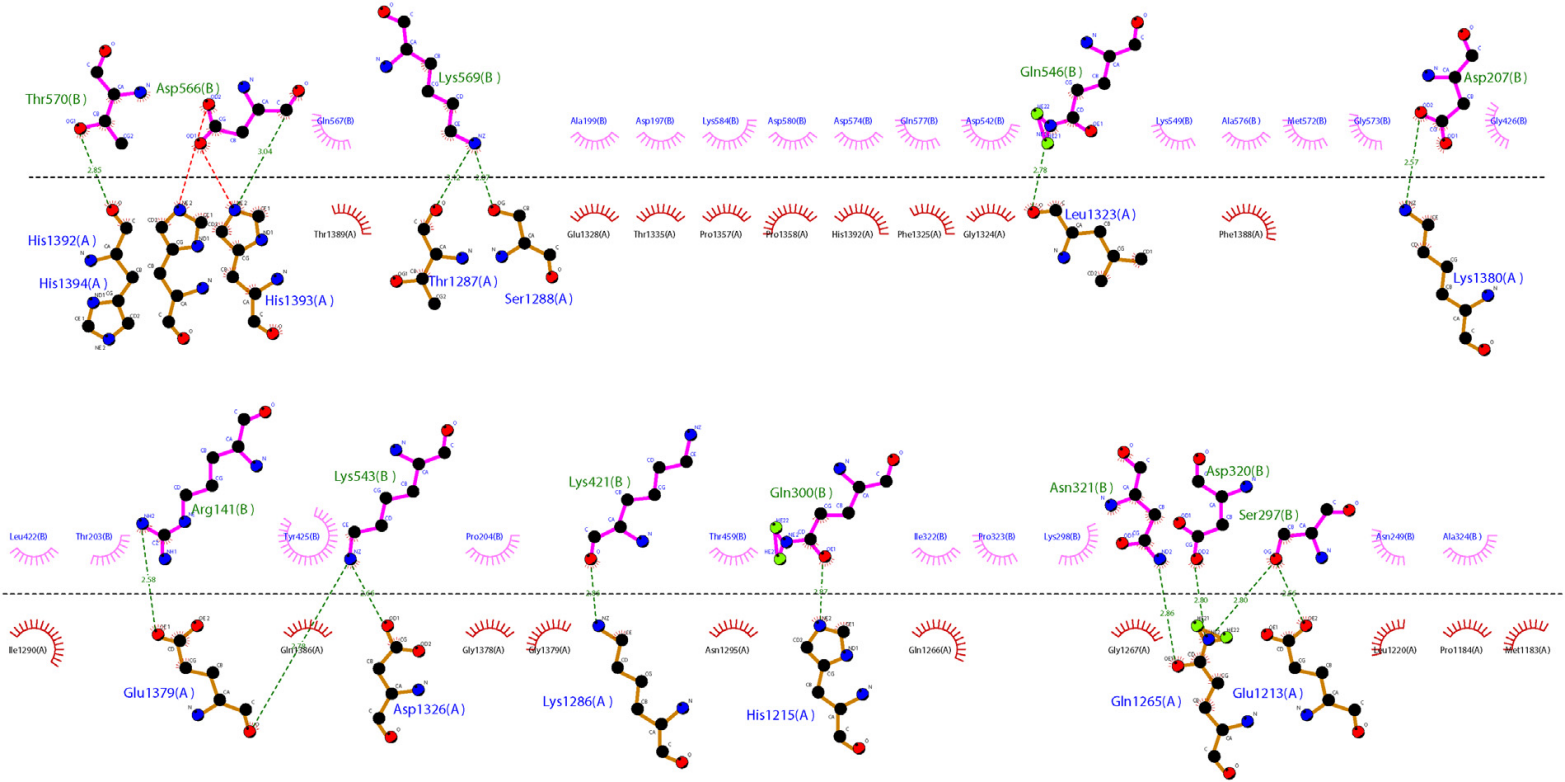
**Ligplot diagram elucidating the protein–protein interactions during molecular docking of CUB7,8 and rat albumin.** Hydrophobic interactions are represented by *red dotted lines* radiating from the amino acids toward the contact atoms. The albumin structure is represented in *purple*, while Cubilin amino acids are shown in *orange*. The possible H-bond interactions between different interacting atoms are represented by *green dotted lines* along with bond lengths. Key amino acids residues are labeled with their position. CUB, complement C1r/C1s, Uegf, Bmp1.

Docking was performed based on XL-MS data by taking account of peptide fragments that were inter protein crosslinked. The top scoring protein-protein docking solution showed interactions of both cubilin (7,8) domains with albumin. Refinement of docking solutions through FireDock showed global interaction energy of −66.63 (contributed by Attractive VdW of −81.8, Repulsive VdW of 21.4, Atomic contact energy of 22.6, and H-bond of −9.5). The cubilin (7,8)-albumin docked complex was stabilized through eleven H-bonds and nine salt-bridge interactions (Fig. 11 and Table S3). With an interface area of 1792.4 Å^2^, −15.2 kcal/mol solvation free energy gain upon interface information was observed indicating a positive protein affinity driven by hydrophobic interactions.

It appears both the domains of cubilin 7,8 are taking part in binding while CUB8 plays a significant role, while in the albumin, DI and DIII domains are taking part in the interaction while DII domain remains untouched. DIII domain of albumin plays a crucial rule based on docking as well as XL-MS and MST data. C terminal region of CUB7,8 appears to be the predominant interacting region as per mass spectrometry data and was further corroborated by docking. Analysis of the binding interface of cubilin 7,8 shows that FcRn and CUB7,8 has distinct binding interface on albumin, although similar albumin domains are involved (DI and DIII). Further structural insights into cubilin protein and albumin complex can only be revealed using high-resolution X-ray diffraction data or CryoEM Data for full-length cubilin and albumin proteins.

### Alterations of cubilin glycosylation in rat model of kidney disease with proteinuria

**Figure 12:**
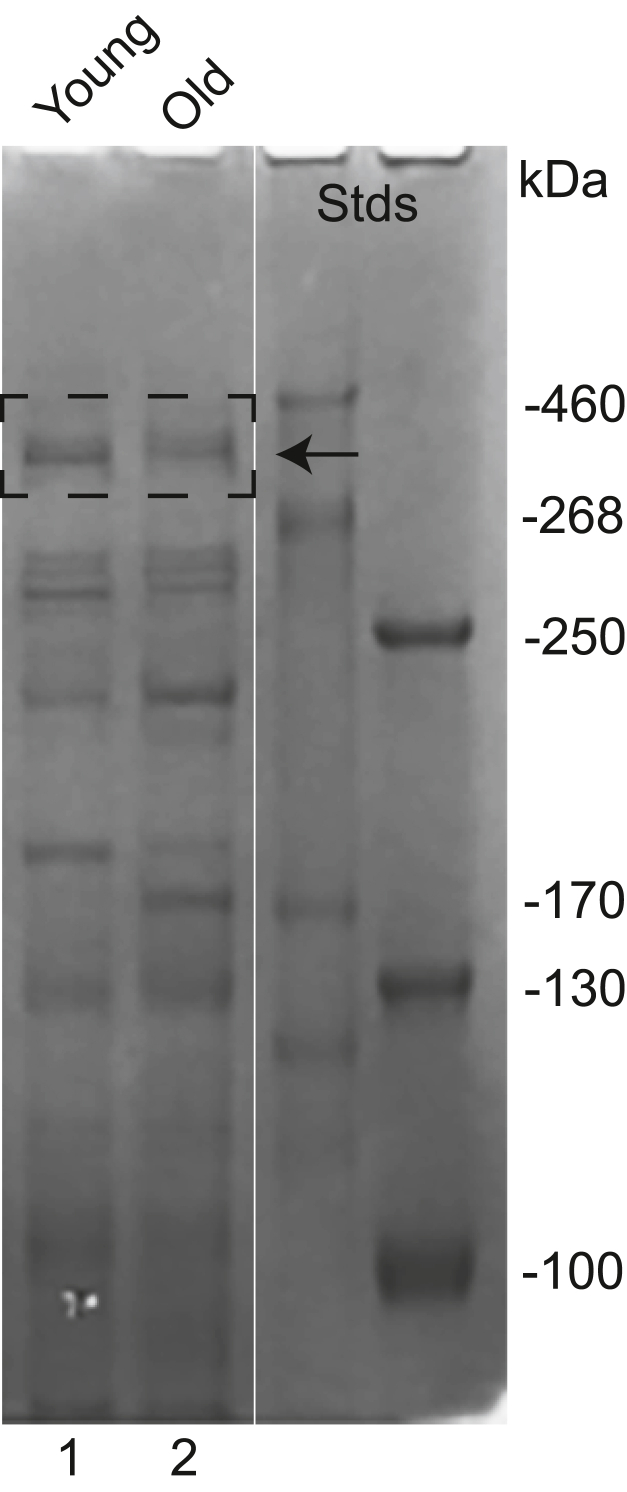
**Isolation of Cubilin from Rat kidney cortex.** Kidney cortex was dissected from both young and old proteinuric MWF rats, homogenized and cubilin enriched. Proteins obtained were separated on 6% SDS-PAGE gels, Coomassie Blue stained and cubilin bands (*dashed box*) show representative band from both young, lane1 and old, lane 2 samples. Two prestained standards were used to better follow the migration and identify bands for analysis. CUB, complement C1r/C1s, Uegf, Bmp1; MWF, Munich Wistar Fromter.

**Figure 13:**
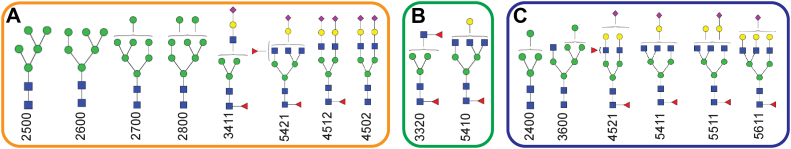
**N-Glycans detected on Cubilin from rat kidney cortex.** N-Glycans identified on peptide CUB8 CDNVVIVN1285K following isolation of cubilin from both young and old rat kidney cortex samples. Glycan structure and 4-digit code are presented for glycans found in both young nonproteinuric rats and old proteinuric rats. The first number in the four-digit code indicates the number of HexNAc, the second number is for Hex, the third is for fucose, and the fourth is for sialic acid. *A*, common glycans attached to site 1285 in CUB8 from both young and old rats; *B*, glycans present only in young rats; *C*, glycans present only in old rats. The complete list of glycans identified for all cubilin peptides and their respective mass and m/z is provided in Table S4. CUB, complement C1r/C1s, Uegf, Bmp1.

Our results show that cubilin glycosylation can impede albumin interaction thus potentially contributing to albuminuria observed in kidney disease. Characterization of purified kidney cortex cubilin’s glycans isolated from young, nonproteinuric Munich Wistar Fromter (MWF) male rats and old, proteinuric MWF male rats identified multiple glycans. Figure 12 shows cubilin-enriched samples separated on a 6% SDS-PAGE gel. Note isolated bands contained 79 ± 7% cubilin, n = 6. Figure 13 shows the glycans detected in the cubilin 8 domain peptide CDNVVIVN1285K with all MS/MS results shown in Table S4. This cubilin 8 domain had the most glycans and the most differences between young and old rat BBM samples. Note, cubilin 8 domain was only found to be glycated at N1285, the site most heavily glycosylated in our 293F cell–expressed protein and closest to the albumin-binding site. Therefore, regulation of glycosylation at N1285 may have direct implications for albumin binding in the PT cells. To evaluate whether any key glycosylation enzymes were altered in the proteinuric animals, a proteomic analysis of kidney cortex was conducted. This analysis identified a 1.6-fold (*p* = 0.02) decrease in phosphoacetylglucosamine mutase (*Pgm3*) protein. *Pgm3* catalyzes the conversion of GlcNAc-6P to GlcNAc-1P, a key step in the hexosamine pathway that results in UDP-GlcNAc which is incorporated into multiple forms of glycosylation (49). Recently, analysis of two *Pgm3* hypomorphic alleles showed significant glycosylation changes in the kidney and a decrease in total serum protein (50). This is also consistent with our analysis of changes in both N & O-glycans analyzed from BBMs (31, 32). Interestingly, our analysis of brush border proteins identified Lamtor5, a member of the Ragulator complex only in the old male proteinuric rats, manuscript under review. The LAMTORs directly impact the activation of mTORC1 and recent data shows this can occur at both the lysosome and Golgi (51, 52). In addition, altered targeting of a glycosyltransferase resulted from an increase in LAMTOR5 (53). It is likely that LAMTOR changes disrupt the normal Golgi organization of glycosyltransferases and glycosidases, the series of glycan enzymes which determine the respective proteins glycan structures reaching the cell surface (54). Further examination of cortex protein changes identified significant fold changes (old proteinuric male/young nonproteinuric male) in a glycosyltransferase B3galnt2 (−1.7) and multiple known endocytic targeting/sorting molecules including Rab5c (−2.0), Rab21 (−1.4), Vamp2 (−1.4), Snx2 (−1.2), and Vps29 (2.3). Note, these protein changes were not detected when comparing old MWF females to MWF young females, data not shown. Table S5 provides the complete list of MWF male kidney cortex proteins (1484) identified and those with *p* < 0.05 differences (254) between young nonproteinuric rats and old hypertensive and proteinuric rats. Fig. S7 presents a Heat Map for the significant protein differences.

## Discussion

Cardiovascular disease and diabetes account for over half the economic cost of noncommunicable diseases worldwide (4). Both diseases increase with age (55, 56). A key contributor to poor health outcomes of cardiovascular disease and diabetes is kidney disease (57, 58, 59). Kidney disease can be defined by reduced kidney function, that is, reduced glomerular filtration rate, and often results in proteinuria or albuminuria (60). Proteinuria increases with age and it is well established that both kidney glomeruli and PTs together determine urine composition and level of proteinuria (5, 61, 62, 63). However, it is the PTs that are first to encounter, sample, and respond to the constituents of the glomerular filtrate (64, 65). To facilitate these important interactions, PT cells have a BBM that increases the surface area for interactions with the glomerular filtrate and contains a unique set of lipids and proteins (66). Importantly, the unique structure of the PT BBM is not present in cell culture models thus emphasizing the importance of *in vivo* and tissue analyses to understand its complexity & function.

Most BBM components, including the multiligand receptors megalin and cubilin, are glycosylated through multiple regulated pathways (67). The diversity of the glycans and their molecular location can impact protein folding, stability, and localization, thus affecting and regulating many biological processes such as cell signaling, adhesion, and communication (68, 69, 70, 71, 72, 73). Consequently, glycan alterations are found in many diseases and multiple studies have identified mutations in megalin and cubilin associated with kidney disease and proteinuria (60, 74, 75, 76, 77, 78, 79, 80, 81, 82, 83, 84, 85, 86, 87, 88, 89, 90, 91, 92). Further, ligand binding of both these multiligand receptors can be impacted by their glycan state which could have a direct impact on levels of proteinuria (16, 17, 28, 30, 93). However, understanding of BBM glycosylation in kidney disease is limited. Thus, since cubilin is the principal albumin-binding receptor in the BBM, a more complete investigation of this interaction and the impact/regulation by glycosylation and disease was the focus of these studies.

The first set of studies focused on investigation of CUB7,8 and CUB6–8, domains sufficient for albumin binding (21). We identified the four primary N-glycosylation sites in CUB7,8 and evaluated the albumin binding impact of mutating the most heavily glycosylated site, N1285P. Blocking glycosylation at N1285 did not result in albumin binding suggesting other glycosylation sites may also influence the albumin interaction possibly by preventing the binding conformation. To investigate a possible pH or Ca^2+^-induced conformation change, similar to what has been shown to occur for low-density lipoprotein receptors (42, 45, 94), TRP fluorescence was evaluated at different pH’s plus or minus Ca^2+^. The magnitude of the change was largest in CUB6–8 and calcium had minimal impact compared to pH. Understanding whether the full-length molecule, containing 27 CUB domains, has even larger shape changes will require further investigation. Cubilin also contains multiple local disulfides that could directly affect ligand binding and understanding whether any reversible will be important (95, 96). Our preliminary data, Table S6, also suggest that glycan trimming by glycosidases other than PNGaseF may enable normal albumin binding. Understanding whether cubilin undergoes dynamic glycosylation in the kidney, BBM could have a direct impact on albumin handling. Future studies should address these possibilities. Altered posttranslational modifications, including glycosylation and carbamylation, are known to be increased by kidney disease resulting in hyperglycemia or hyperuremia as occurs in diabetes and chronic kidney disease respectively (31, 32, 97, 98, 99, 100, 101).

Our next set of studies sought to better define the structural conformation of CUB7,8, CUB6–8, and their association with albumin. These studies used molecular modeling, SAXS, the amine to amine crosslinker, BS3, to better define the binding site between albumin and cubilin.

**Figure 14:**
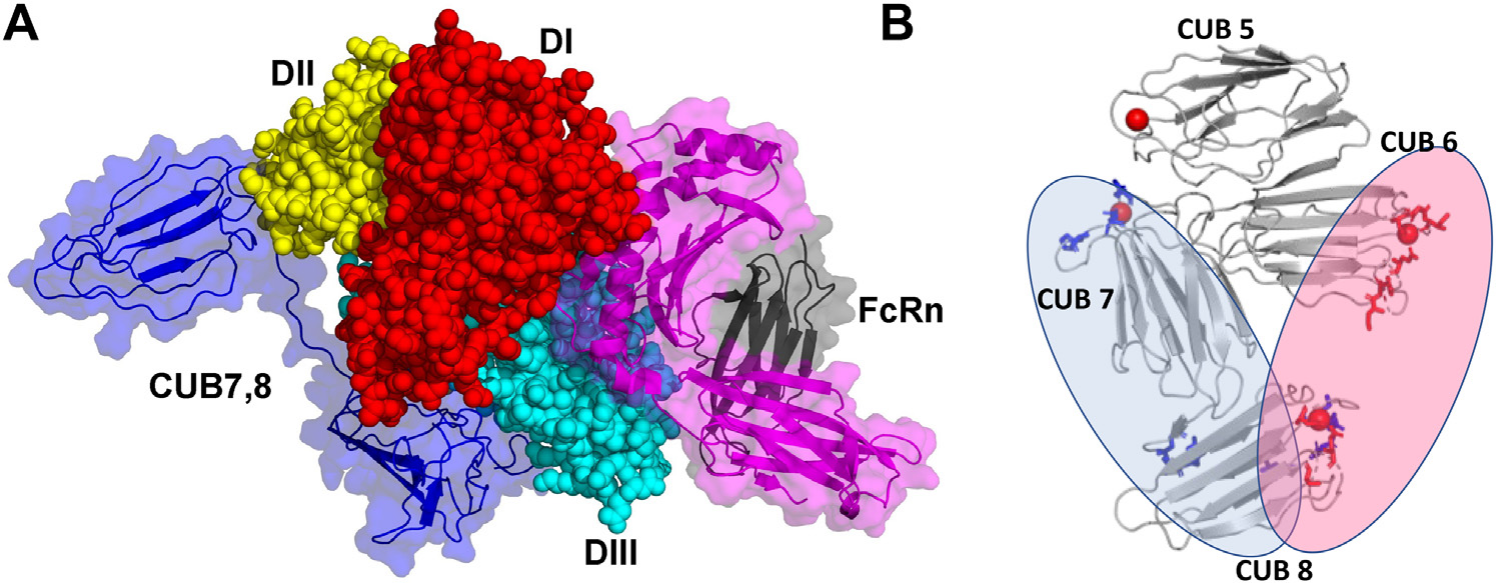
**Binding interfaces of albumin-binding receptors/ligands.***A*, distinctive binding interface of FcRn (PDB ID 4N0F) and CUB7,8 (our results) with albumin, showing DIII domain as common binding interface. *B*, figure showing binding interface of albumin (our results, *blue color*) and Intrinsic factor (IF-B12, PDB ID 3KQ4, *red color*) with cubilin. CUB, complement C1r/C1s, Uegf, Bmp1; IF, intrinsic factor.

SEC-MALS-SAXS was performed to reveal the dynamic shape of albumin bound to CUB domains. Inline SEC-MALS, in addition to SAXS, confirmed the solution behavior of CUB domains having some self-associating amino acid sequences (as per *in silico* and MS analysis) but maintaining predominantly monomeric behavior in solution. Surprisingly after mutation of a key glycosylation site, CUB7,8 N1285P mutant shows oligomeric behavior confirmed in SEC-MALS and SEC-SAXS, pointing toward the importance of glycosylation in binding and maintaining the integrity of domains. BS3 crosslinking followed by XL-MS revealed significant amino acids involve in interaction, these interactions were used in computational docking to depict the putative binding interface. These interactions are likely affected by the glycosylation state and may confer stearic hindrance, thus having a pivotal role in regulating albumin interactions. These studies combined enable us to propose the following model predicting the binding interface necessary for cubilin-albumin binding, Figure 14.

Finally, we asked whether in a disease state could we identify altered glycans at the N1285 site which we documented to be critical for albumin binding. Results suggest that cubilin isolated from proteinuric rats contains more complex glycans at N1285 site, which may reflect altered glycan trimming due to changes in glycosidase levels, activity, or location. Preliminary analyses of other glycosidases support that incomplete trimming of glycan can restore binding between the expressed cubilin domains and albumin. In addition, proteomic analysis of kidney cortex proteins identified a decrease in Pgm3 in the proteinuric animals, a key enzyme in the pathway of glycosylation. Further investigation of the impact of glycan changes on cubilin in regards to interacting with its ligands (*i.e.*, albumin, megalin) and their potential impact on its BBM structure and associations (8, 102) are necessary to better understand the dynamic uptake of albumin by the kidney PTs.

In summary, we have identified amino acids on the cubilin domains that interact with albumin and identified glycosylation sites that impact this interaction. In addition, we show structurally why glycosylation at N1285 blocks *in vitro* binding and show that this site is altered in a rat model of proteinuria. Future work will target regulatory pathways that may dynamically control cubilin glycosylation and/or conformation thus directly impacting albumin binding and ultimately levels of proteinuria. In addition, evaluation of albumin-cubilin binding will better define PT endocytosis mechanisms for this important serum protein and determine whether alternative endocytic mechanisms are responsible for modified albumins, that is, glycated, carbamylated as encountered in multiple disease states including diabetes and kidney disease (30, 35).

## Experimental procedures

### Proteins

Expressed proteins had their sequence optimized using Life Technologies or Genscript services. Respective sequences were cloned into the pcDNA3.4 mammalian expression vector that contains the CMV promoter. Protein expression took place in the Gibco Expi293F expression system using the Expi293F cells according to the manufacturer’s protocol. Protein sequences of each rat cubilin domain and albumin DIII domain are shown in Fig. S1. Each cubilin protein contained a C-terminus 6X histidine tag. Cubilin proteins were purified using a Ni-NTA Superflow column (Qiagen) followed by Gel Filtration on a Toyopearl HW55S column. Albumin domain III was purified using Blue Sepharose followed by gel filtration as for cubilin. RSA was purchased from Sigma. Figure 2 shows a Coomassie Blue–stained SDS-PAGE gel of purified albumin domain III (DIII), cubilin 7,8 (CUB7,8), and cubilin 6–8 (CUB6–8). Glycated and MGO albumin was prepared as described previously (35). Briefly, albumin was incubated at 37 °C for 21 days in the presence of glucose (20, 200, and 500 mM) to make RSA20G, RSA200G, and RSA500G or MGO (1 and 5 mM) to make RSA1MGO and RSA5MGO.

### Cubilin deglycosylation

CUB7,8 and CUB6–8 were deglycosylated with PNGaseF (New England Biolabs, NEB or Bulldog Bio) in the presence of 0.5% NP-40, 0.05 M Sodium Phosphate pH 7.5 at 37 °C.

### Microscale thermophoresis

MST was used to characterize the binding affinity of Cubilin domains (CUB7,8 and CUB6–8) with albumin (full length and domain III). Binding assays were performed with the Monolith NT.115 MST device using standard capillaries (NanoTemper Technologies) (103, 104). Measurements were performed at 25 °C in 67 mM NaPO_4_ buffer, 150 mM NaCl, and 0.05% Tween 20 at pH 6.0 or pH 7.4 + 1 mM CaCl_2_. The infrared laser power was between 20 and 60%, and 40 to 70% LED power was used. A laser on time of 30 s and a laser off time of 5 s was used. Data from a minimum of three replicate binding assays were analyzed using Nanotemper analysis, Origin 9.0 (https://www.originlab.com) and GraphPad Prism 9.3 software (https://www.graphpad.com/scientific-software/prism/). Albumins were conjugated with a fluorescent tag [Texas Red-X succinimidyl ester, (AAT Bioquest) or Oregon Green 488-X succinimidyl ester, (ThermoFisher)], using standard procedures to achieve a dye:protein molar ratio of 1:1 as described previously (35). Note, cubilin binding to albumin was the same with either Texas Red-X succinimidyl ester-albumin or Oregon Green 488-X succinimidyl ester-albumin conjugate. However, as we have reported for the albumin–FcRn interaction, decreased binding was observed at >1:1 dye:albumin conjugation ratios (35). The impact of cubilin glycosylation, buffer pH, and [Ca^2+^] on albumin binding were also evaluated as described later.

### Intrinsic tryptophan fluorescence of cubilin domains

Purified CUB7,8, CUB6–8, and buffers had Ca^2+^ removed by using Chelex resin as described by Reese and Moss (44). Proteins were then buffer exchanged and concentrated using Amicon spin concentrators into either pH 5.0 (50 mM Citrate, 100 mM KCl), pH 6.0 (50 mM Citrate, 100 mM KCl), or pH 7.5 (10 mM MOPs, 100 mM KCl) buffers. CaCl_2_ was added to 1 mM for + Ca^2+^ samples and all samples were placed in a Greiner UVStar microplate (#675801) at a concentration of 2 mg/ml. A fluorescence emission scan from 340 to 360 nm, 1 nm intervals (filter for excitation was ex280-10 with a long pass dichroic filter of LP310) was done using the Clariostar plate reader (BMG LabTech), n = 3.

### Analysis of cubilin glycosylation

#### Sample preparation

CUB7,8 gel bands ± PNGaseF treatment and cubilin gel bands from young and old rat kidney cortex were processed with in-gel digestion to extract peptides and glycopeptides. Briefly, gel bands were washed with ddH_2_O, cut into 1 mm cubes, then washed sequentially with ddH_2_O, 50% acetonitrile (ACN), and 100% ACN, three times. Next, gel bands were washed with 50 mM ammonium bicarbonate and 50% ACN, followed by gel drying. For alkylation steps, 10 mM DTT was added to two gel samples and incubated at 56 °C for 45 min. After incubation, solutions were discarded and 55 mM iodoacetamide was added with 30 min incubation in the dark. Gels were washed with 50 mM ammonium bicarbonate and 50% ACN, respectively. For digestion steps, trypsin solution was added to two dried gel samples followed by incubation for 45 min on an ice bath. The solution above the gels was pulled off and 50 mM ammonium bicarbonate buffer was added. Gel bands were incubated at 37 °C overnight. After incubation, 1% formic acid was added to deactivate trypsin and supernatant was collected. The remaining peptides were extracted with 30%, 60%, and 90% ACN with 0.1% formic acid under sonication, respectively. Peptides and glycopeptides extracted from CUB7,8 gel bands without PNGaseF digestion were separated into two portions. One was for proteomic analysis and the other one was for glycomic analysis. The second portion of the sample was treated with PNGaseF with incubation at 37 °C for 18 h to release glycans. Released glycans were reduced and permethylated following the previously described protocol (105). Gel bands containing full length cubilin derived from young and old rat kidney cortex were digested with the addition of chymotrypsin.

#### Method description for the analysis of cubilin glycosylation

Samples were analyzed with the Dionex 3000 UltiMate NanoLC system (Dionex) coupled with LTQ Orbitrap Velos mass spectrometers (Thermo Scientific). In the LC system, a reverse-phase C18 trap (Acclaim PepMap 100, 75 μm × 2 mm, 3 μm, 100 Å, Thermo Scientific) was used for purification. A C18 column (Acclaim PepMap 100, 75 μm × 150 mm, 2 μm, 100 Å, Thermo Scientific) was used to separate peptides, glycopeptides, or glycans. The gradient and mass spectrometry methods set up for proteomic and glycomic study were performed as previously described (32, 106).

#### Data analysis method

The proteomic raw files were converted to an mgf. file by Proteome Discover software (Version 1.2, Thermo Scientific) and then searched against a RatUniprot database in MASCOT (Version 2.4, Matrix Science). MASCOT parameters were set as follows: fixed modification of carbamidomethylation on cysteine and variable modification of oxidation of methionine. Trypsin/chymotrypsin as the enzyme and a maximum of two missed cleavages were selected. The peptide tolerance was set to 10 ppm. The MS/MS tolerance was set to 0.8 Da. MultiGlycan software (Version 1.4) (https://bio.tools/multiglycan) was used to search for possible glycan structures followed by a manual check (107). The confirmation of *N*-glycan structures was based on checking the full MS and MS^2^ manually using Xcalibur (Thermo Scientific) software. The identification of glycopeptides was confirmed by manual checking the full MS and MS^2^.

### MALDI-TOF analysis of protein molecular weight

The molecular weight of purified proteins and deglycosylated proteins were determined using a 4800 MALDI TOF/TOF analyzer (AB SCIEX) equipped with a pulsed Nd: YAG laser at an excitation wavelength of 355 nm. One thousand two hundred fifty laser shots were fired (50 sub-spectra, 25 shots per sub-spectrum) for each MS spectrum. MALDI-spectra were recorded in positive-ion mode. Laser intensity was set to 4000 to 5000. The mass spectra were analyzed using Data Explorer 4.9 software (AB SCIEX). Samples were spotted and mixed with an equal volume of the sample and sinapinic acid matrix on a MALDI plate. Sinapinic acid was dissolved in 50% ACN/49.9% water/0.1% TFA at a concentration of 50 mM as the MALDI matrix.

### Crosslinking of CUB7,8 and albumin

The equimolar ratio of PNGaseF deglycosylated CUB7,8 and RSA were mixed and incubated for 10 min at 30 °C to enable binding (binding buffer = 67 mM NaPO_4_ buffer, 150 mM NaCl, 1 mM Ca^2+^, 0.05% Tween 20 at pH 7.4). Crosslinking was initiated by the addition of 25 mM crosslinker BS3 to a final concentration of 2 mM. Crosslinking reaction was performed at 30 °C for 20, 30, or 60 min. The reaction was quenched by the addition of 1 M Tris pH 8.0 to 50 mM. Samples were loaded on 4 to 20% Tricine SDS-PAGE and Coomassie Blue stained gel bands removed for Mass spectrometry.

### Analysis of crosslinked peptides

#### Sample preparation method

Gel bands were processed with in-gel digestion protocol as described previously. Each gel band was divided into two samples for LC-MS/MS analysis. One was digested with trypsin only, the other one was digested with trypsin followed by GluC digestion (18 h incubation at 37 °C). All peptides extracted from the gel bands were collected, dried, and resuspended with 2% ACN, 98% water, and 0.1% FA. The gradient and mass spectrometry methods were conducted as previously described (106). Samples were analyzed by Q Exactive HF (Thermo Scientific) and LTQ Orbitrap Velos mass spectrometers.

#### Data analysis method

Raw files were subjected to pLink software (Version 2.3.8-Institute of Computing Technology, Chinese Academy of Sciences) to identify the BS3 cross-linked peptides (108). Parameters were set up as follows: flow type-conventional crosslinking; process number = 2; linker = BS3. Trypsin or Trypsin+GluC were chosen for enzymes. Up to three missed cleavages were chosen. Peptide mass range was 600 to 6000, and peptide length was 6 to 60 amino acids. The precursor tolerance was ±20 ppm, and fragment tolerance was ±20 ppm. Fixed modification of carbamidomethylation on cysteine and variable modification of oxidation of methionine were chosen. Filter tolerance was ±10 ppm. The separate false discovery rate was less than 5% at the peptide-spectrum match level.

### Sequence retrieval, model building, docking, and evaluation

Protein sequence of rat cubilin seventh domain (CUB7:1165–1277) and cubilin eighth domain (CUB8:1278–1389) of full length cubilin UniProtKB ID O70244 (Cubn_RAT) were used in this work. Previous studies supported that CUB7,8 bound albumin (21). For the current study, a homology-based model of the rat CUB7,8 domains as a receptor for docking was generated using the Phyre2 loop modeling server in extensive mode. Sequence for the ligand, rat albumin, was retrieved from UniprotKB ID P02770 and a similar method was applied to obtain its model.

Modeling of cubilin–albumin complex was done using Haddock2.4 (High Ambiguity Driven biomolecular DOCKing) webserver (109). The active site residues were chosen based on the residue interaction network obtained from XL-MS. The top solutions were further refined using FireDock (110). Interfacial protein-protein interacting was calculated with PDBPisa (111).

### Analysis and visualization

Analysis of the interactions of the docked complex was done by PDBsum using LigPlot^+^ v. 1.4 (112). It automatically analyses interactions mediated by hydrogen bonds and hydrophobic contacts. The cleft analysis tool in PDBsum was employed to calculate the cleft size. The PyMOL Molecular Graphics System was used to visualize various poses of docked proteins and generated images (113).

#### Equilibration SAXS

Equilibration SAXS data was collected prior to beamline SAXS data collection at Anton Paar Virginia. These measurements were done to optimize the buffer conditions and concentrations optimal for SEC-SAXS data collection. The data was recorded on SAXSpoint 2.0 equipped with Primux 100 Cu k-α and Eiger 1M detector and TC150 stage. Data was reduced by fitting beam zero and masking beamstop, finally integration of data to create 1D curve.

### Size-exclusion chromatography and multi angle light scattering

The Agilent Infinity II HPLCs each with a Wyatt DAWN HELEOS II MALS+DLS (17 channels LS, plus 1 DLS) detector and a Wyatt Optilab T-rEX dRI detector was used for the characterization of samples through MALS. In all instances, UV absorption spectroscopy data were recorded at 280 nm. The MALLS system was calibrated relative to the light scattering of toluene for an absolute RI measurement of the mobile phase. The differential RI increment, dn/dc (ml/g), of each protein sample was calculated from the primary amino acid sequence using the method of Zhao *et al.* (114), which is integrated into the SEDFIT ‘vbar and dn/dc calculator’ considering the experimentally determined RI of the solvent, measurement temperature (25 °C), and RI laser wavelength (658 nm). The molecular weight estimates, MWMALLS, were determined from the three-angle MALLS scattering intensities combined with the protein concentration determined from RI through the SEC elution peak of each sample using the ASTRA 7 software package (Wyatt Technology Corporation). In addition, the integrated QELS detector was used to evaluate the hydrodynamic radius, RH, of the protein samples in the mobile phase, with the incorporation of a correction for solvent viscosity due to the effect of 2% v/v glycerol in the running buffer. The viscosity of the 2% v/v glycerol running buffer was estimated at 0.9476 cP using the ‘Calculate density and viscosity of glycerol/water mixtures’ calculator based on an assumption that glycerol, and not the other buffer components, is primarily responsible for affecting the translational diffusion coefficient of the proteins in solution compared to its value in water.

### Size exclusion chromatography and small angle X-ray scattering

SEC-SAXS was performed at BioCAT (beamline 18ID at the Advanced Photon Source) with in-line size-exclusion chromatography. Samples in buffer (67 mM NaPO_4_ buffer, 150 mM NaCl, and 0.05% Tween 20 at pH 6.0 or pH 7.4 + 1 mM Ca^2+^) were loaded onto an equilibrated Superdex 200 10/300 GL column, which was maintained at a constant flow rate of 0.7 ml/min using an AKTA Pure FPLC (GE Healthcare Life Sciences) and the eluate after it passed through the UV monitor was directed through the SAXS flow cell, which consists of a 1 mm ID quartz capillary with 5 mm walls. A coflowing buffer sheath was used to separate the sample from the capillary walls, helping to prevent radiation damage. Scattering intensity was recorded using a Pilatus3 1M (Dectris) detector which was placed 3.5 m from the sample giving access to a q-range of 0.004 to 0.4 Å^−1^. A series of 0.5 s exposures were acquired every 2 s during elution and data was reduced using BioXTAS RAW 1.6.3 (115). Buffer blanks were created by averaging regions flanking the elution peak and subtracting from exposures selected from the elution peak to create the I(q) *versus* q curves used for subsequent analyses. More information on SAXS beamline and data collection parameters and data analysis software is provided in Table S1.

### SAXS models generation and analysis

The intensity files obtained from BioXTAS RAW 1.6.3 were analyzed further using ATSAS 3.0 data analysis software for small-angle scattering data analysis from biological macromolecules (116). In ATSAS/Primus window, click on radius of gyration function to calculate Rg and Rc derived from Guinier approximation in low q region. Distance distribution function is used to calculate the maximum linear dimension Dmax of the scattering species. Dammif function was used for creating SAXS models, further refined by Dammin and averaged by damaver function of ATSAS/Primus. Averaged SAXS models were then superimposed using supcomb function over the homology model of CUB7,8 and Cub6–8. For visualization and figure generation, PyMOL software was utilized.

#### Animals

Tissue (kidney cortex) was collected from male MWF rats and frozen in LN2 till cubilin isolation. MWF rats (9–12 weeks old) were derived from a colony generously provided by Dr Roland Blantz (UCSF) and maintained in the Indiana University LARC facility. MWF rats have been studied extensively and are distinguished by the development of hypertension and albuminuria by week 8 in the males which escalated progressively to ≥400 mg/24 h urinary albumin excretion by week 32, with approximately 50% sclerotic glomeruli by week 40 (5, 117, 118). Note, females do not develop hypertension or proteinuria as they age. All experiments followed NIH Guide for the Care and Use of Laboratory Animals guidelines and were approved by the Animal Care and Use Committee at the Indiana University School of Medicine.

#### Cubilin purification

The method (102) was adapted to provide an enriched sample that could be separated on SDS-PAGE allowing cubilin band analysis by mass spectrometry. Briefly, kidney cortex was homogenized in 20 volumes (ml/g) of Tris-buffered saline pH 7.5 + protease inhibitors using a Brinkman polytron (PTA10S) followed by centrifugation for 20 min, 4 °C, 12,000*g*. Pellet underwent repeat homogenization and centrifugation and was then resuspended by dounce homogenization in five volumes (ml/g) of TBS buffer pH 7.5 + 10 mM EDTA. Suspension was rotated for 30 min to release cubilin followed by repeat centrifugation. A second EDTA resuspension and centrifugation were performed and EDTA supernatants pooled. EDTA supernatant was diluted 2:1 with H_2_O and added to DEAE-Sepharose Fast Flow beads (1 ml/g of tissue) equilibrated in TRIS buffer pH 7.5 + EDTA. After rotation for several hours or overnight, beads were pelleted and supernatant collected and adjusted to pH 8.0 by addition of NaOH. Q-Sepharose Fast Flow beads (1 ml/g tissue) were equilibrated in TRIS buffer pH 8.0 + EDTA and added to DEAE supernatant and rotation repeated, cubilin binds to Q-Sepharose. After rotation, beads were washed with Q equilibration buffer and cubilin eluted by addition of buffer + 0.5 M NaCl. Elution was concentrated using an Amicon Ultra 4 10kd centrifugal filter and gel sample made by diluting 1:1 with sample buffer (119) (8 M Urea, 2 M Thiourea, 0.05 M Tris (pH 6.8), 30% glycerol, 75 mM DTT, 3% SDS, 0.05% bromophenol blue) and heating at 60 °C for 10 min. Protein separation was achieved using 6% Novex WedgeWell Tris-Glycine gel. To follow migration and identify molecular weight of bands, two prestained molecular weight standards were used (1. PageRuler Plus pre-stained protein ladder #26619, Thermo Scientific and 2. Hi Mark prestained protein standards #LC5699, Life Technologies).

## Data availability

All data are included within this article.

## Supporting information

This article contains supporting information (37).

## Conflict of interest

The authors declare that they have no conflicts of interest with the contents of this article.
