# Supplementary material for: Glycosylation of a key cubilin Asn residue results in reduced binding to albumin
Source: J Biol Chem. 2022 Aug 13;298(10):102371. doi: 10.1016/j.jbc.2022.102371 (PMC9485058; doi:10.1016/j.jbc.2022.102371)
Supplement: Supplemental Table S1 [file mmc1.docx]

| **BioCAT SAXS Beamline and data collection parameters, ANL Chicago** | |
| --- | --- |
| **Instrument** | BioCAT Beamline 18ID, ANL, Chicago |
| **Detector type and model** | Pixel, Pilatus3 1M Dectris detector |
| **Monochromators** | Si <111> and <400> |
| **Wavelength Å** | 1.033 |
| **Q range (Å^-1^)** | .004-0.4 |
| **Goniometer** | 0.3m - 3.5 m SAXS instrument |
| **Angular Resolution (µrad^2^ FWHM)** | 160 x 190 |
| **Energy Resolution (dE/E)** | 2 x 10^-4^ (Si <111>) |
| **Minimum Spot Size (µm^2^ FWHM)** | < 1500 x 3500 (unfocused),  ~30 x 150 (focused at detector 3.5 m from sample) |
| **Temperature** | 283 K |
| **Sample mounting** | Quartz capillary |
| **Software Employed and data analysis** | |
| **Primary data reduction** | BioXTAS RAW 1.4.0 (reference) |
| **Data processing** | SAS Data Analysis (ATSAS 2.8.4) |
| **Ab initio analysis** | DAMMIF/DAMMIN |
| **Symmetry, anisotropy assumptions** | P1 , none |
| **Validation and averaging** | Refined DAMMIN model |
| **Mw determination** | DATMOW |
| **Computation of model intensities** | CRYSOL |
| **3D Graphics representation** | PyMOL |

**Table S1**
