# Supplementary material for: Glycosylation of a key cubilin Asn residue results in reduced binding to albumin
Source: J Biol Chem. 2022 Aug 13;298(10):102371. doi: 10.1016/j.jbc.2022.102371 (PMC9485058; doi:10.1016/j.jbc.2022.102371)
Supplement: Supplemental Table S3 [file mmc3.docx]

**Table S3**

**Hydrogen bonds and salt-bridge interactions**

**between CUB7,8 and albumin**

**H-bonds**

| 1 | A:GLN 1264 [HE21] | 2.19 | B:ASP 320 [OD2] |
| --- | --- | --- | --- |
| 2 | A:GLN 1264 [HE22] | 2.09 | B:SER 297 [OG] |
| 3 | A:LYS 1286 [HZ2] | 2.18 | B:LYS 421 [O] |
| 4 | A:LYS 1380 [HZ2] | 1.56 | B:ASP 207 [OD2] |
| 5 | A:GLN 1264 [OE1] | 2.49 | B:ASN 321 [HD21] |
| 6 | A:THR 1287 [O] | 2.49 | B:LYS 569 [HZ1] |
| 7 | A:SER 1288 [OG] | 2.01 | B:LYS 569 [HZ3] |
| 8 | A:LEU 1323 [O] | 1.80 | B:GLN 546 [HE21] |
| 9 | A:ASP 1326 [OD1] | 1.88 | B:LYS 543 [HZ2] |
| 10 | A:GLU 1379 [OE1] | 1.53 | B:ARG 141 [HH21] |
| 11 | A:GLU 1379 [O] | 2.03 | B:LYS 543 [HZ1] |

**Salt-bridges**

| 1 | A:LYS 1380 [NZ] | 3.48 | B:ASP 207 [OD1] |
| --- | --- | --- | --- |
| 2 | A:LYS 1380 [NZ] | 2.57 | B:ASP 207 [OD1] |
| 3 | A:HIS 1393 [NE2] | 3.01 | B:ASP 566 [OD1] |
| 4 | A:HIS 1393 [NE2] | 3.39 | B:ASP 566 [OD2] |
| 5 | A:HIS 1394 [NE2] | 2.90 | B:ASP 566 [OD1] |
| 6 | A:HIS 1394 [NE2] | 2.73 | B:ASP 566 [OD2] |
| 7 | A:ASP 1326 [OD1] | 2.66 | B:LYS 543 [NZ] |
| 8 | A:GLU 1379 [OE1] | 2.58 | B:ARG 141 [NH2] |
| 9 | A:GLU 1379 [OE1] | 3.44 | B:ARG 141 [NH1] |
