# Supplementary material for: Glycosylation of a key cubilin Asn residue results in reduced binding to albumin
Source: J Biol Chem. 2022 Aug 13;298(10):102371. doi: 10.1016/j.jbc.2022.102371 (PMC9485058; doi:10.1016/j.jbc.2022.102371)
Supplement: Supplemental Table S6 [file mmc6.pdf]

Table S6

Albumin binding to Cubilin constructs treated with different glycosidases

Cubilin construct	Glycosidase	Binding affinity (kD)
CUB7,8	PNGaseF	<1μM
	Mannosidase (NEB P0768S)	<1μM
CUBN140P	PNGaseF	<1μM
	Mannosidase (NEB P0768S)	<1μM
	Fucosidase (NEB P0748S)	<1μM
	Neuraminidase A (P0722S)	<1μM
CUB6-8	PNGaseF	<1μM
CUB6-8	Mannosidase (NEB P0768S)	<1μM

All assays used the MST instrument and a fluorescent conjugated Albumin. Increased or no binding was observed if deglycosylation took place in the absence of NP-40. These preliminary studies support the exciting possibility of glycan modulation to regulate cubilin albumin interaction.
