# Supplementary material for: Glycosylation of a key cubilin Asn residue results in reduced binding to albumin
Source: J Biol Chem. 2022 Aug 13;298(10):102371. doi: 10.1016/j.jbc.2022.102371 (PMC9485058; doi:10.1016/j.jbc.2022.102371)
Supplement: Supplemental Figure S1 [file mmc7.pdf]

Supporting Information

Figure S1

Cubilin 7,8 & 6-8 constructs for 293F cell expression

Rat Cubilin 7,8 & 6-8 each have preprotrypsin signal peptide MSALLILALVGAAVA-

Cubilin 7,8 Purified protein has this sequence, 232 AA, Mass (Da):26,253

1165-CGGNLT TPTGVLTSPN YPMPYYHSSE CYWRLEASHG
SPFELEFQDF HLEHHPSCSL DYLAVFDGPT TNSRLIDKLC GDTTPAPIRS
NKDVVLLKLR TDAGQ QGRGF EINFRQRCDN VVIVNKTSGI LESINYPNPY
DKNQRCNWTI QATTGNTVNY TFLGFDVESY MNCSTDYVEL YDGPQWMGRY
CGNNMPPPGA TTGSQLHVLF HTDGINSGEK GFKMQWFTHG HHHHHH

Rat Cubilin 6-8 Purified protein has this sequence, 349 AA, Mass (Da):39,340

1048-CLYDYTDNFGM LSSPNFPNNY PSNWECIYRI TVGLNQQIAL
HFTDFTLEDY FGSQCVDFVE IRDGGYETSP LVGIYCGSVL PPTIISHSNK LWLKFKSDAA
LTAKGFSAYW DGSSTGCGGN LTTPTGVLTS PNYPMPYYHS SECYWRLEAS HGSPFELEFQ
DFHLEHHPSC SLDYLAVFDG PTTNSRLIDK LCGDTTPAPI RSNKDVVLLK LRTDAGQQGR
GFEINFRQRC DNVVIVNKTS GILESINYPN PYDKNQRCNW TIQATTGNTV NYTFLGFDVE
SYMNCSTDYV ELYDGPQWMG RYCGNNMPPP GATTGSQLHV LFHTDGINSG
EKGFKMQWFT HG HHHHHH

Rat Albumin Domain III Purified protein has this sequence, 204 AA, Mass (Da): 22,723

405-VEEPKN LVKTNCELYE KLGEYGFQNA VLVRYTQKAP QVSTPTLVEA
ARNLGRVGTK CCTLPEAQRL PCVEDYLSAI LNRLCVLHEK TPVSEKVTKC
CSGSLVERRP CFSALTVDET YVPKEFKAET FTFHSDICTL PDKEKQIKKQ
TALAELVKHK PKATEDQLKT VMGDFAQFVD KCCKAADKDN CFATEGPNLV
ARSKEALA
