# Supplementary material for: Glycosylation of a key cubilin Asn residue results in reduced binding to albumin
Source: J Biol Chem. 2022 Aug 13;298(10):102371. doi: 10.1016/j.jbc.2022.102371 (PMC9485058; doi:10.1016/j.jbc.2022.102371)

<u>Figure S2</u>- Molecular weight determination of CUB7,8 and CUB6-8 by MALDI-MS before and after PNGaseF deglycosylation.

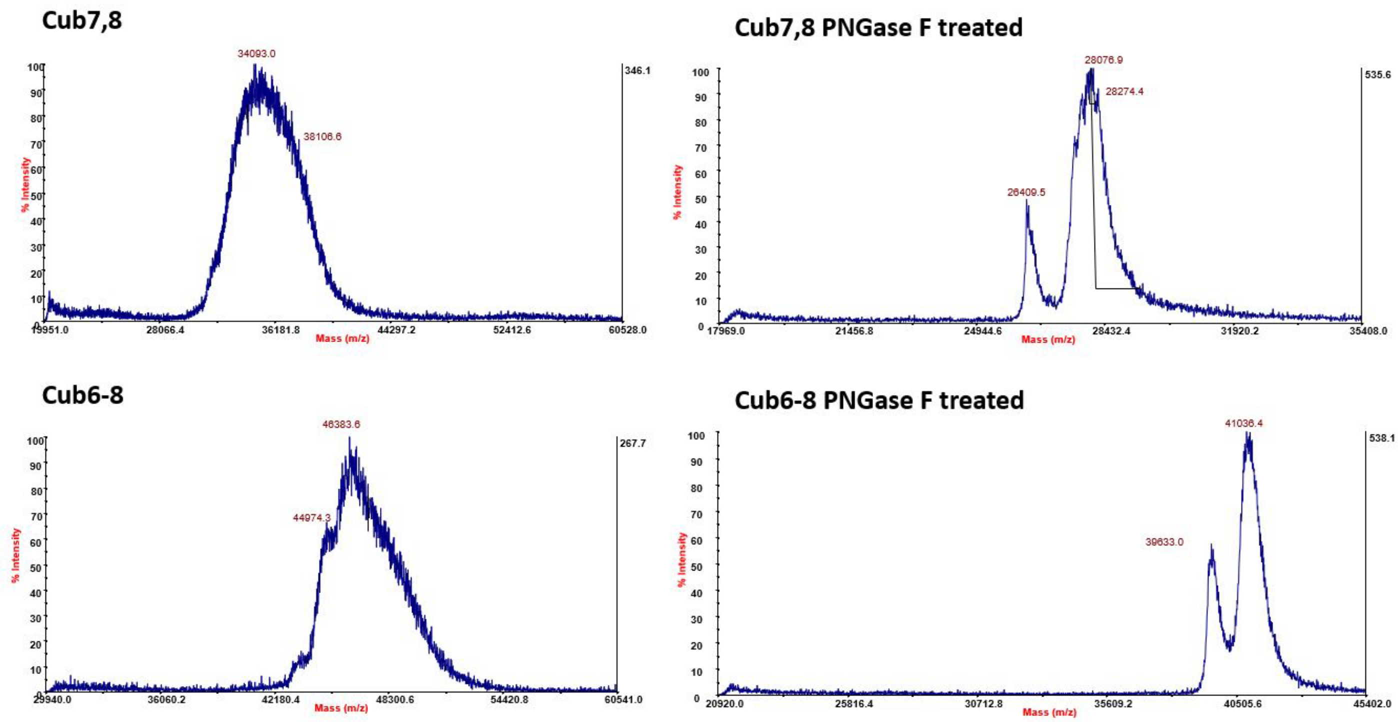

Supplement: Supplemental Figure S2 [file mmc8.pdf]
