# Supplementary material for: Glycosylation of a key cubilin Asn residue results in reduced binding to albumin
Source: J Biol Chem. 2022 Aug 13;298(10):102371. doi: 10.1016/j.jbc.2022.102371 (PMC9485058; doi:10.1016/j.jbc.2022.102371)
Supplement: Supplemental Figure S3 [file mmc9.pdf]

Figure S3

Unknown modifications of CDNVVIVNK

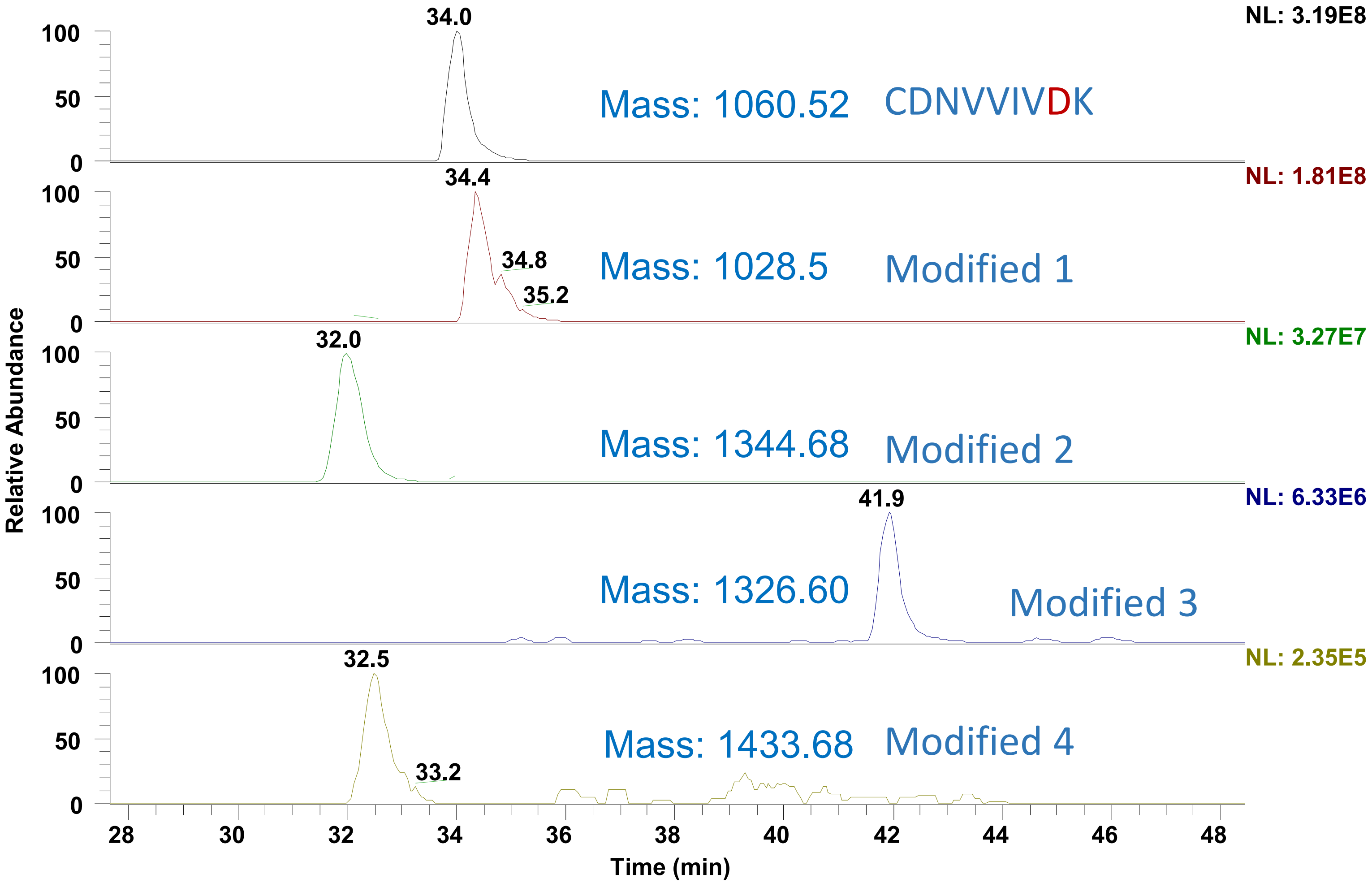

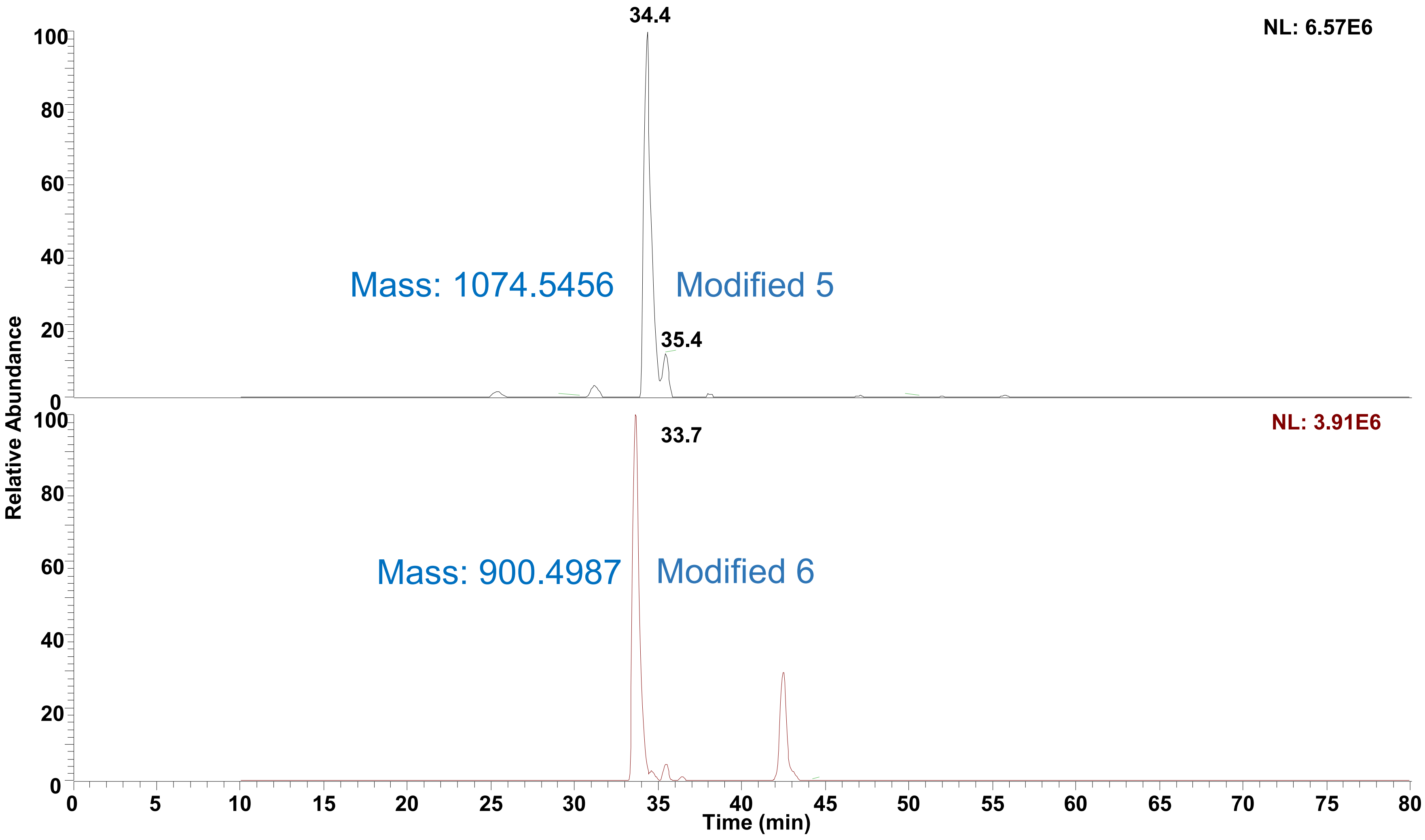
34.4
NL: 6.57E6
Mass: 1074.5456
Modified 5
35.4
NL: 3.91E6
33.7
Mass: 900.4987
Modified 6
Relative Abundance
Time (min)
0
5
10
15
20
25
30
35
40
45
50
55
60
65
70
75
80
100
80
60
40
20
0

Figure S3

Unknown modifications of CDNVVIVNK confirmed at MS/MS level

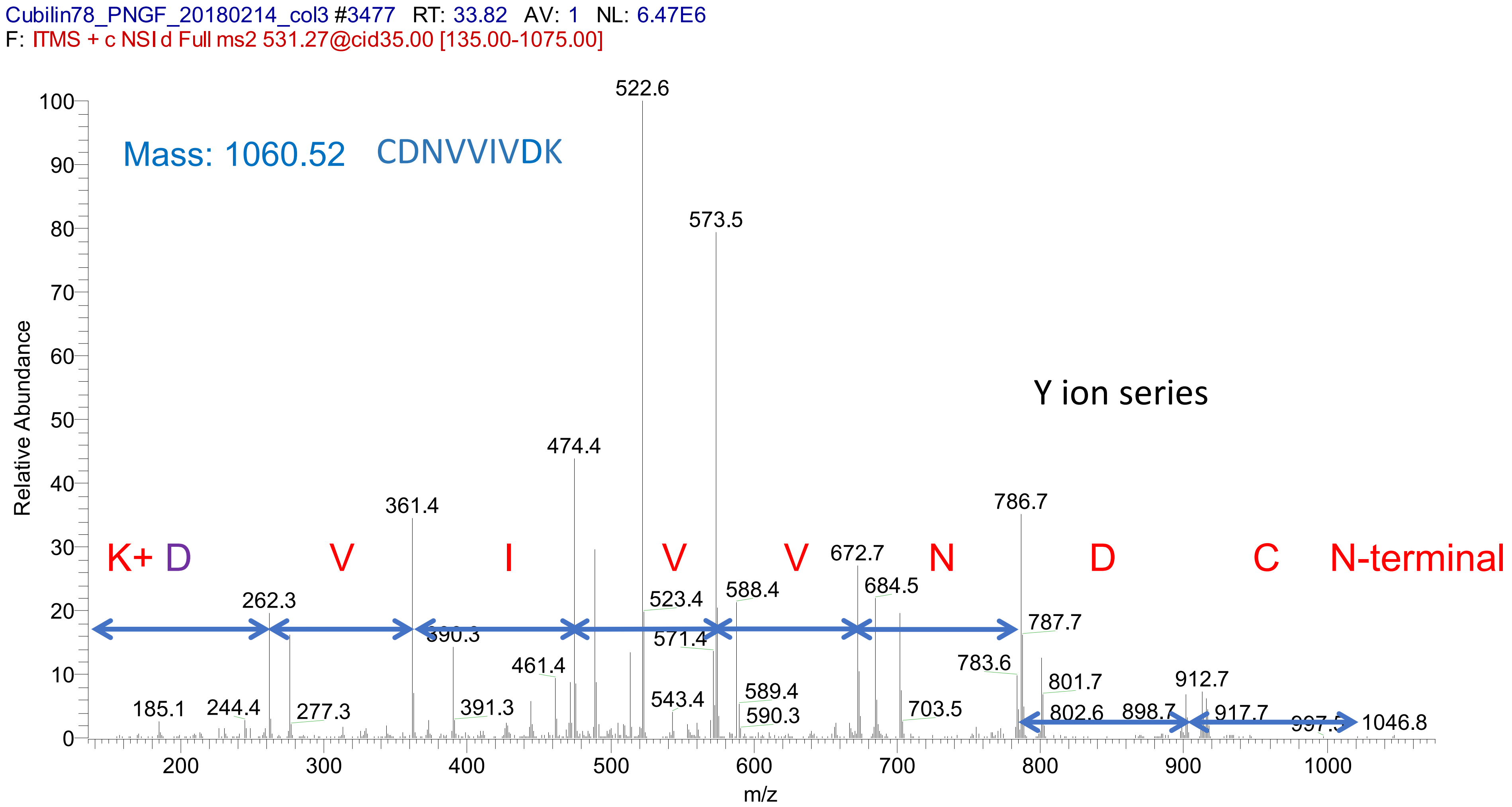

Figure S3

Unknown modifications of CDNVVIVNK confirmed at MS/MS level

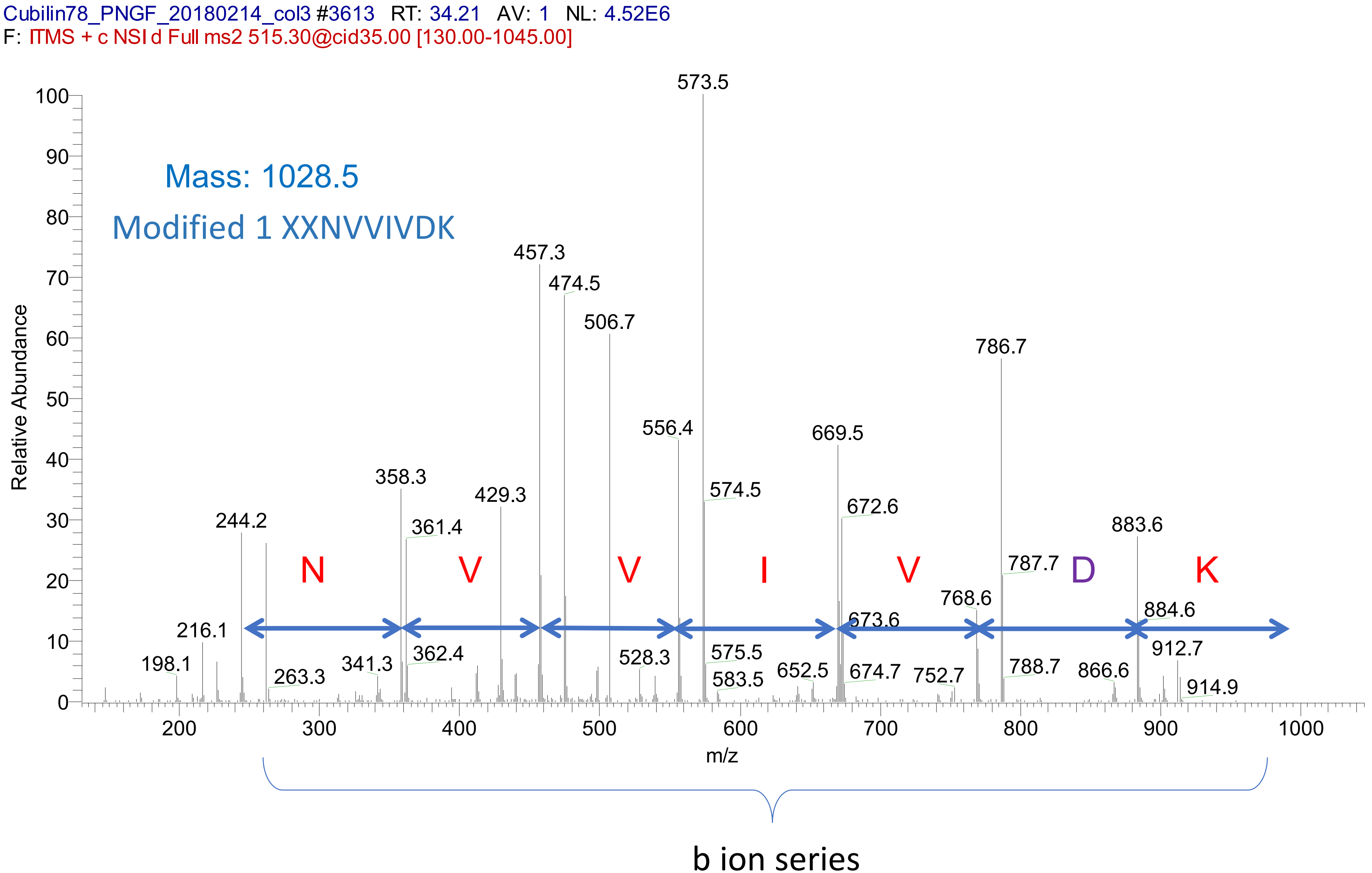

Figure S3

Unknown modifications of CDNVVIVNK confirmed at MS/MS level

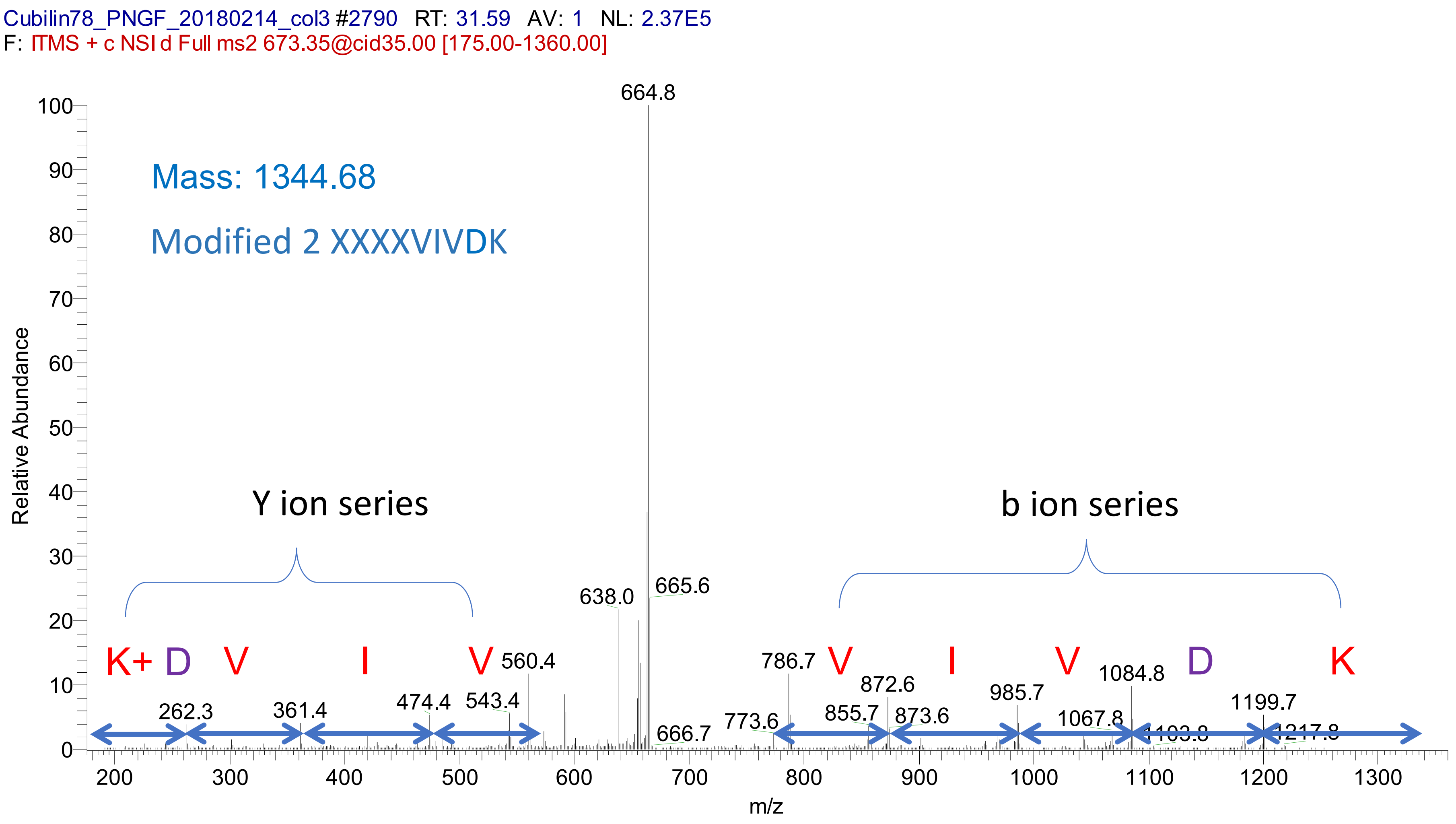

Figure S3

Unknown modifications of CDNVVIVNK confirmed at MS/MS level

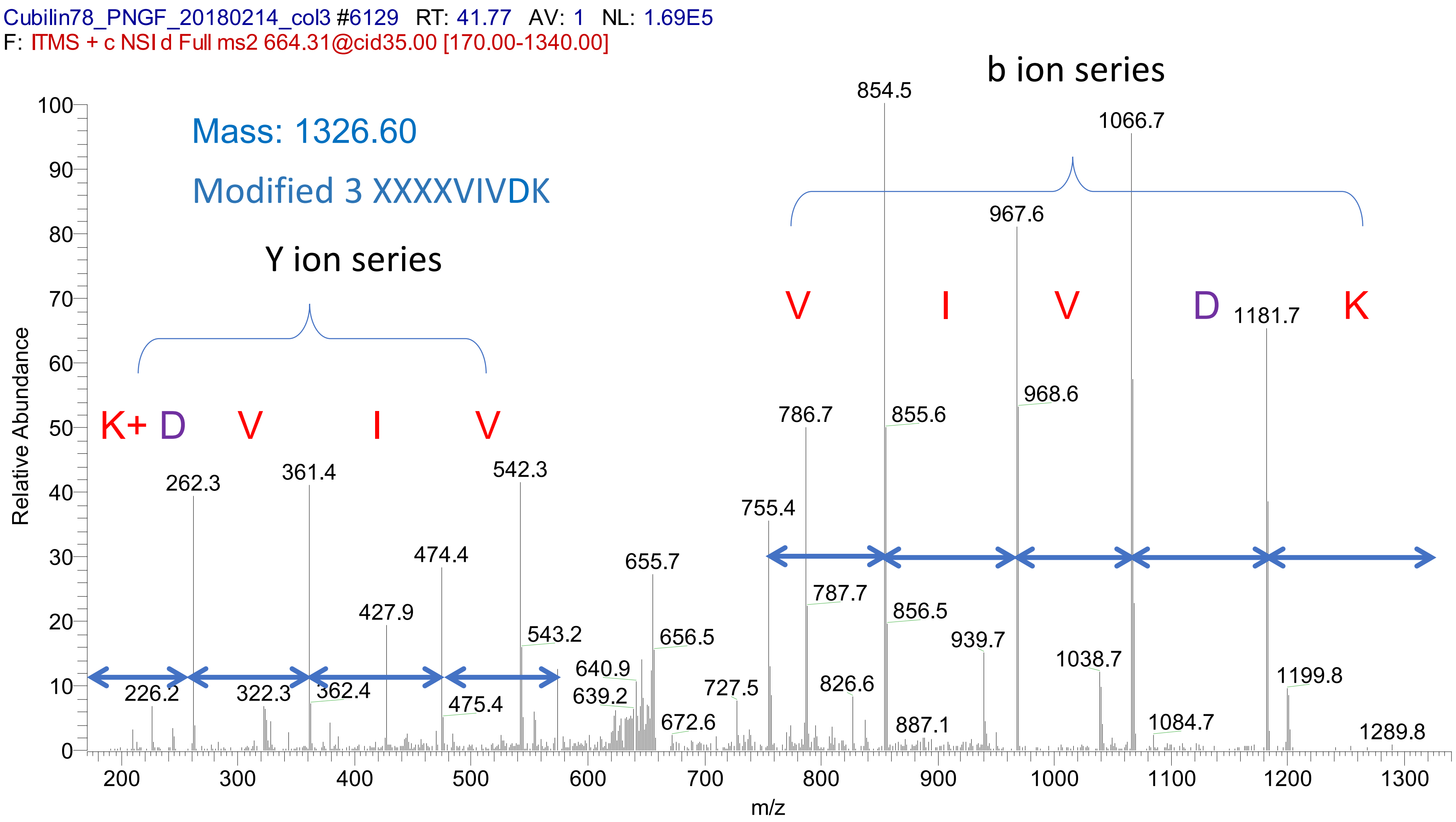

Figure S3

Unknown modifications of CDNVVIVNK confirmed at MS/MS level

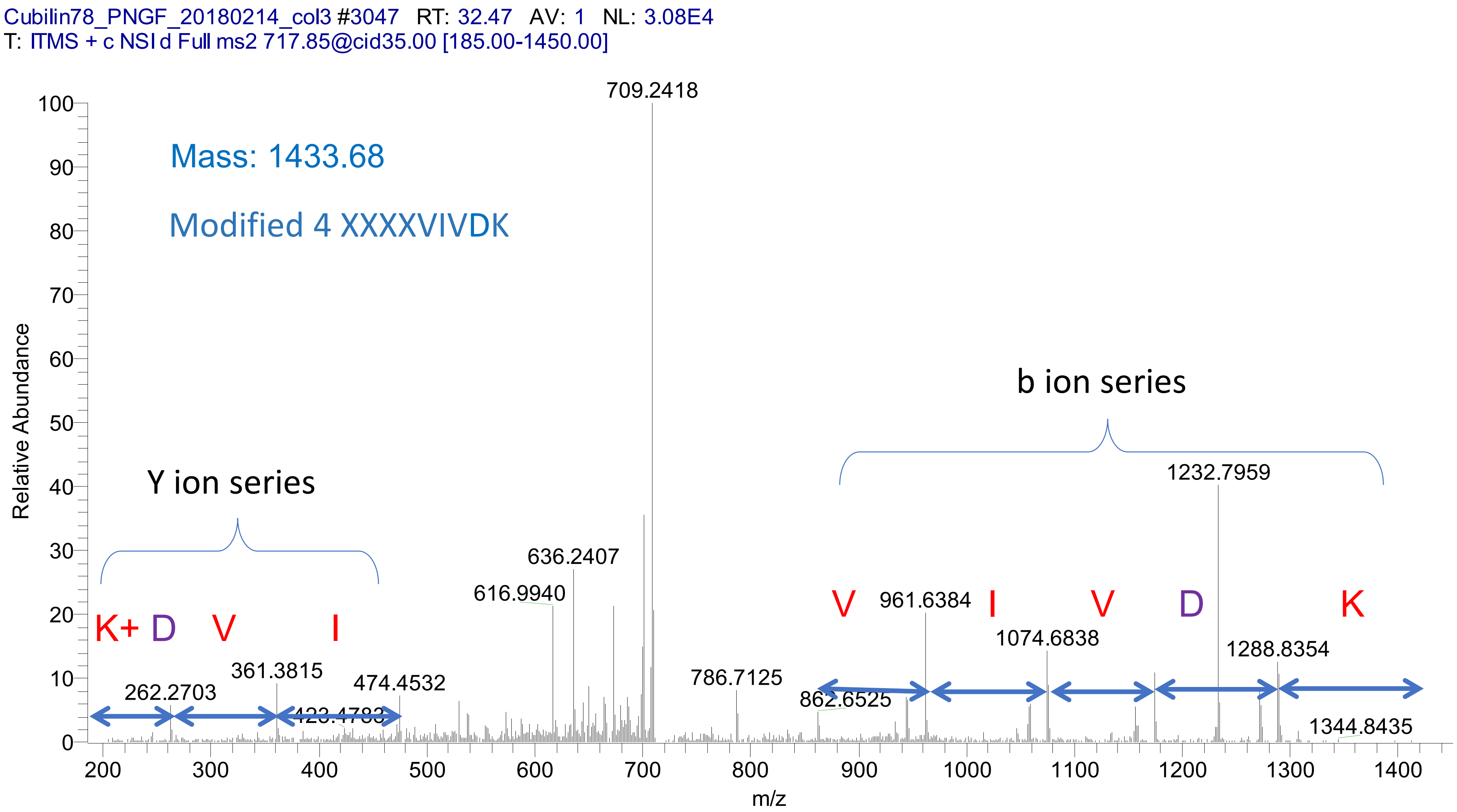

Figure S3

Unknown modifications of CDNVVIVNK confirmed at MS/MS level

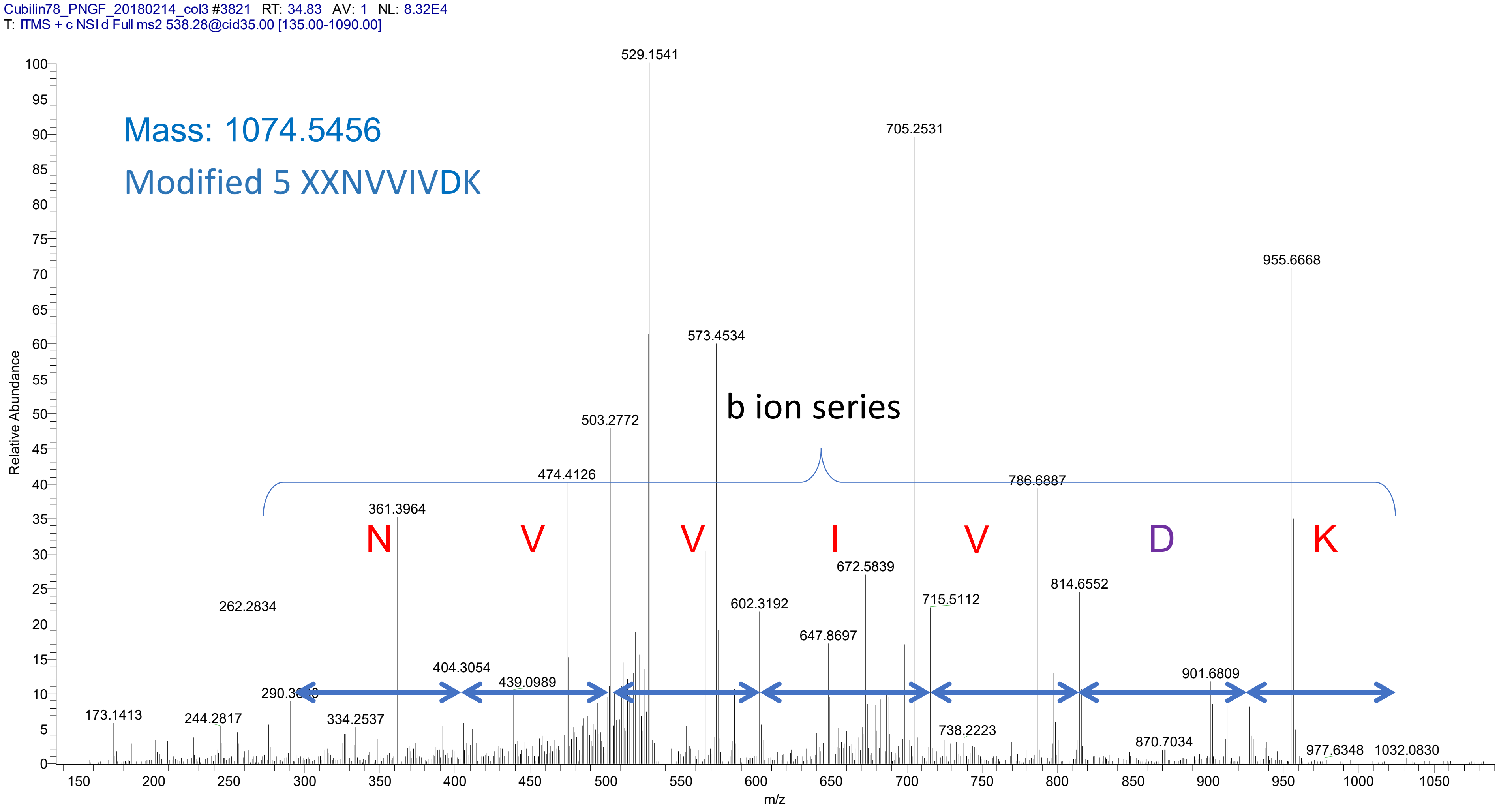

Figure S3

Unknown modifications of CDNVVIVNK confirmed at MS/MS level

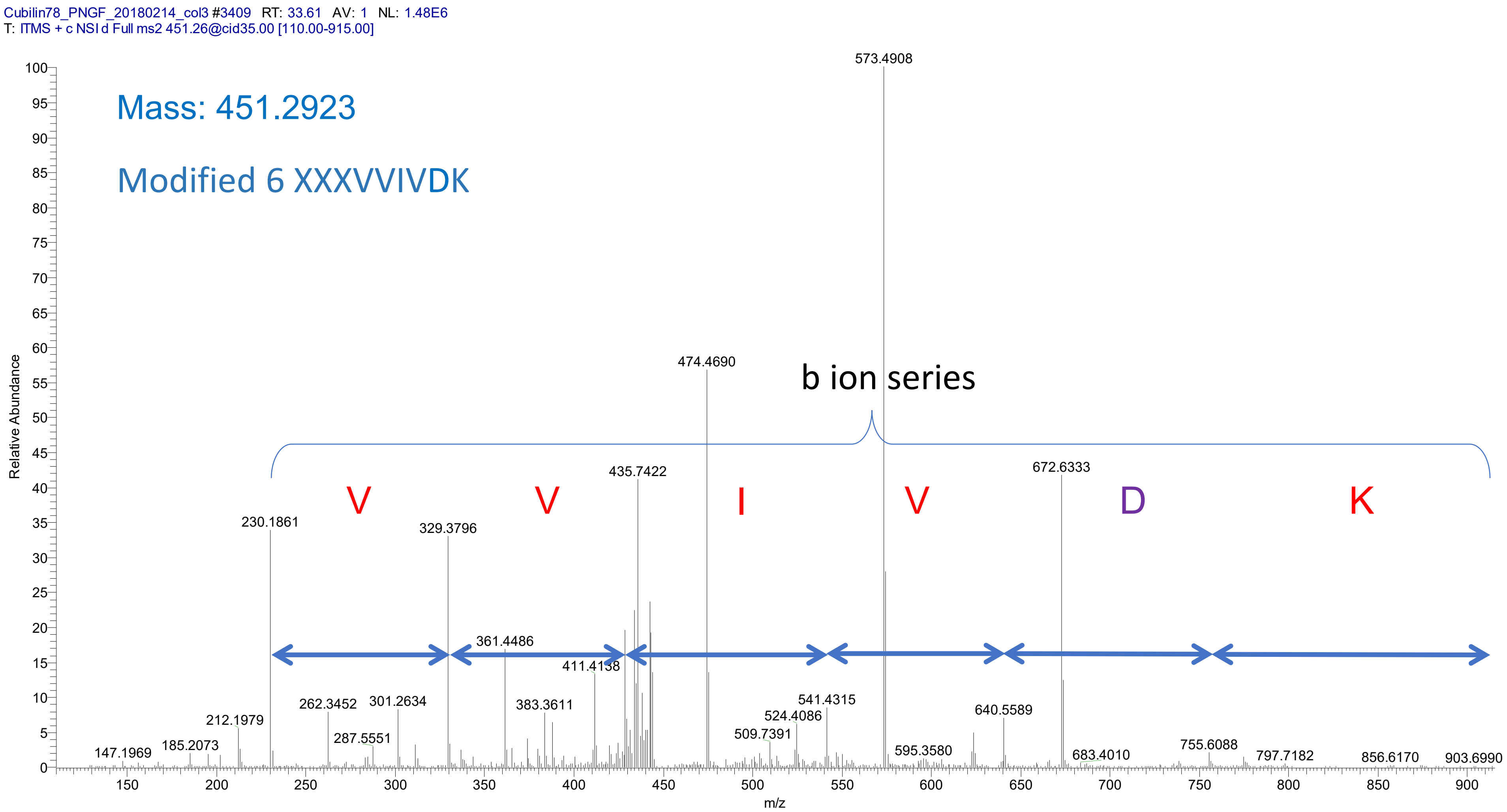

Figure S3

O-glycopeptides identified in Cub 7,8 samples (detected in untreated and PNGase F digested samples)

m/z	Glycan Structure	Peptide Mass
856.4086	HexNAc1Hex1Fuc1	1199.6125
702.3510	HexNAc1	1199.608
783.3774	HexNAc1Hex1	1199.608
928.9246	HexNAc1Hex1NeuAc1	1199.607

Figure S3

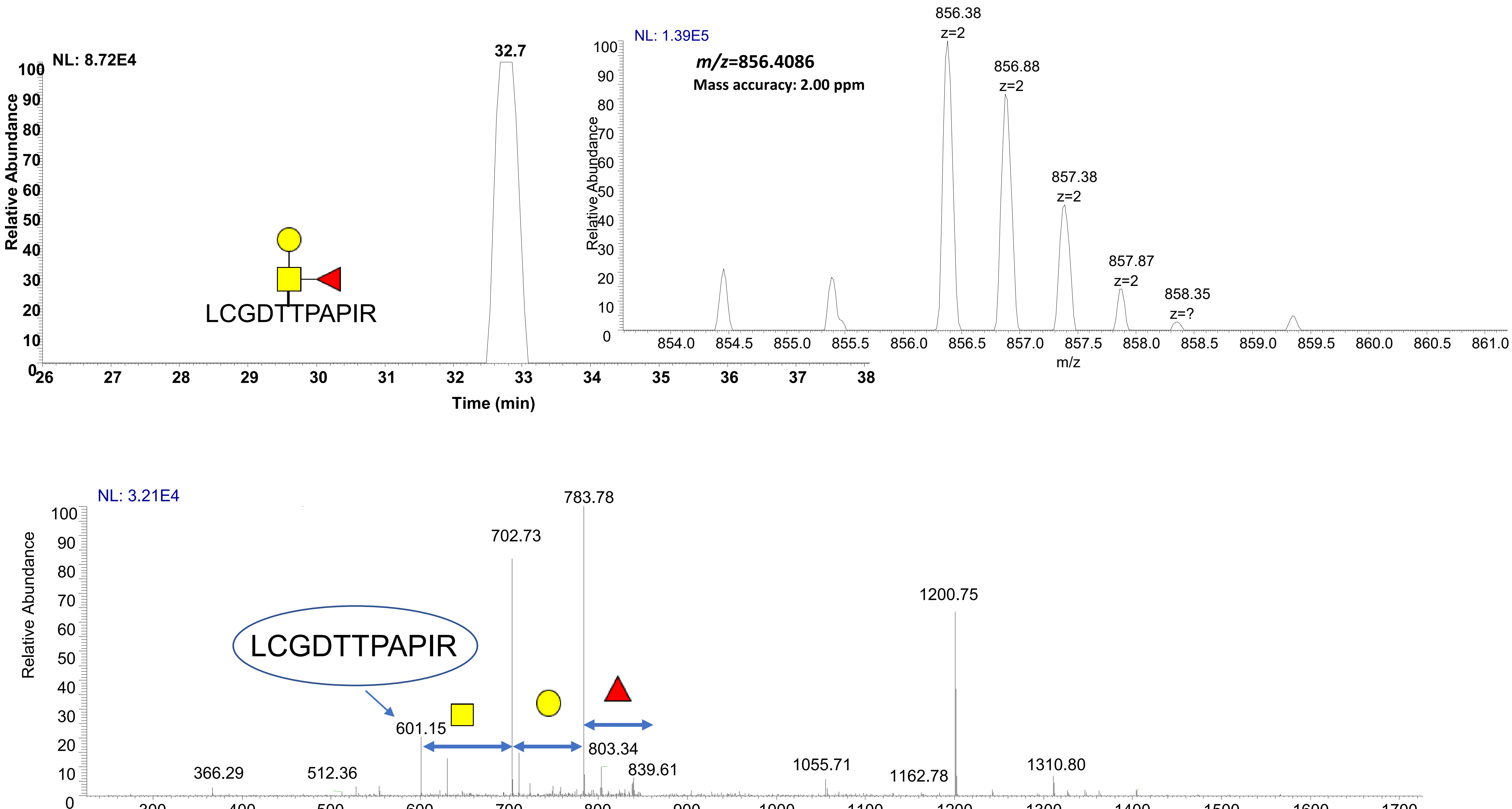
NL: 8.72E4
32.7
Relative Abundance
LCGDTTPAPIR
Time (min)
NL: 1.39E5
m/z=856.4086
Mass accuracy: 2.00 ppm
856.38
z=2
856.88
z=2
857.38
z=2
857.87
z=2
858.35
z=?
m/z
NL: 3.21E4
783.78
702.73
1200.75
LCGDTTPAPIR
601.15
803.34
839.61
366.29
512.36
1055.71
1162.78
1310.80
m/z
Figure S3

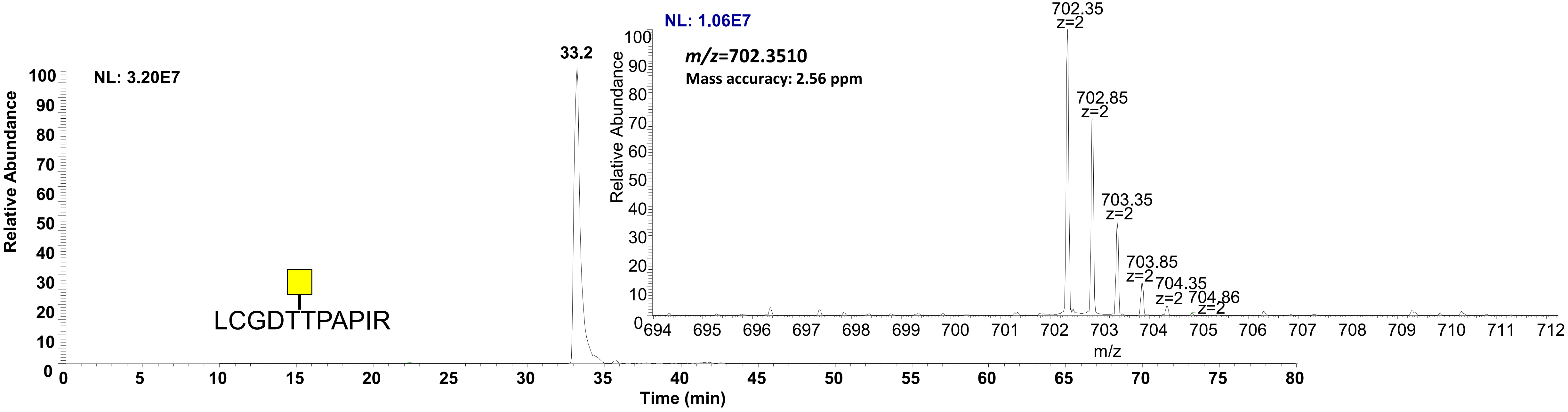
NL: 3.20E7
33.2
LCGDTTPAPIR
Relative Abundance
Time (min)
NL: 1.06E7
m/z=702.3510
Mass accuracy: 2.56 ppm
702.35 z=2
702.85 z=2
703.35 z=2
703.85 z=2
704.35 z=2
704.86 z=2
m/z

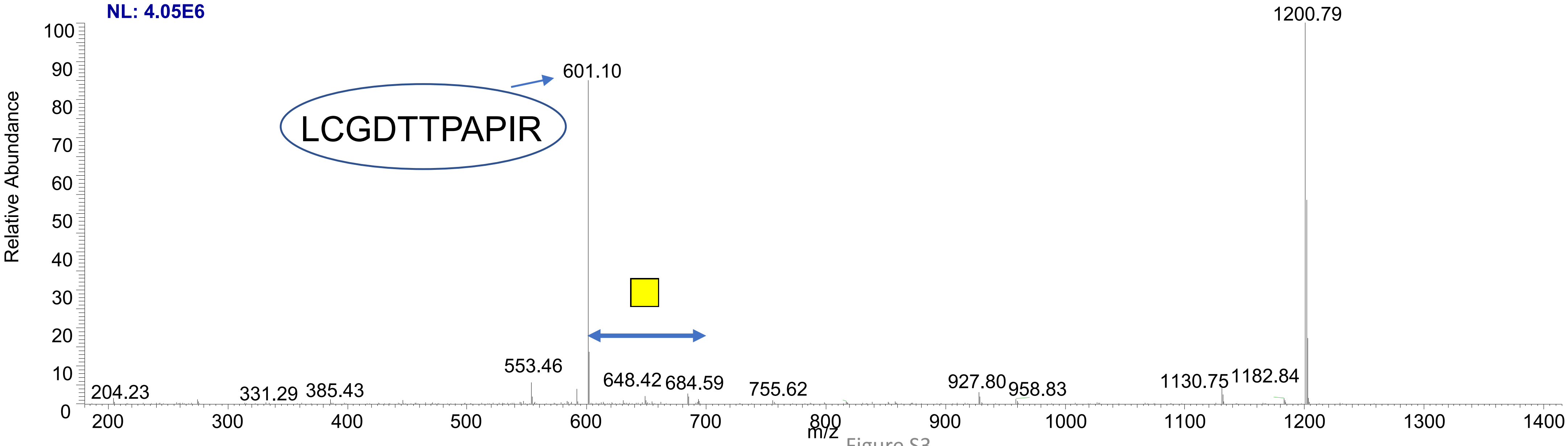
NL: 4.05E6
LCGDTTPAPIR
601.10
1200.79
553.46
204.23
331.29
385.43
648.42
684.59
755.62
927.80
958.83
1130.75
1182.84
Relative Abundance
m/z

Figure S3

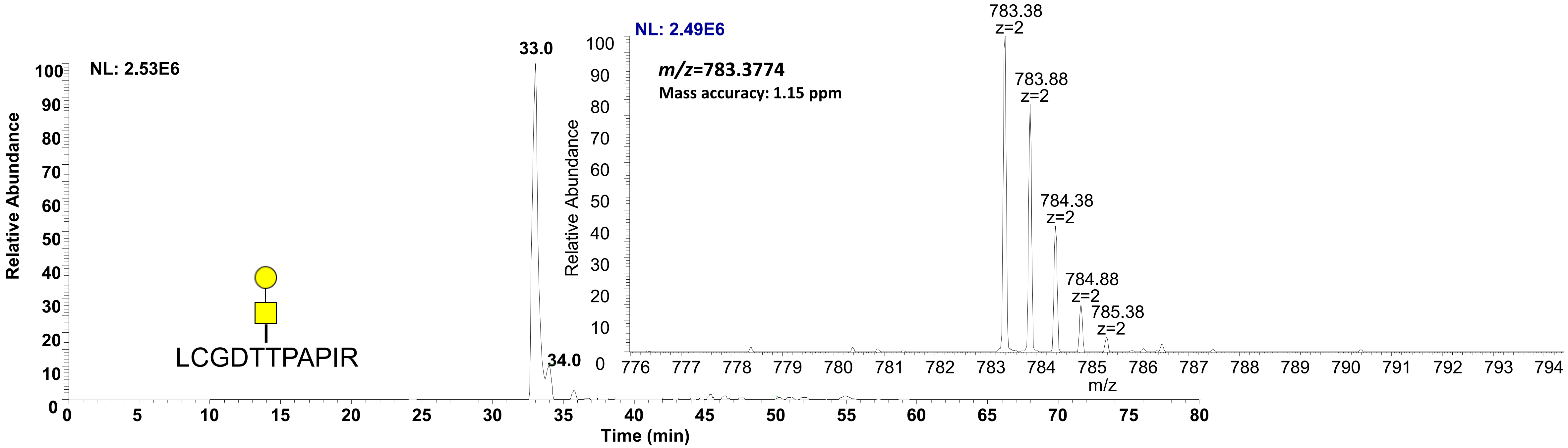

NL: 2.53E6
33.0
34.0
LCGDTTPAPIR
Relative Abundance
Time (min)
NL: 2.49E6
m/z=783.3774
Mass accuracy: 1.15 ppm
783.38
z=2
783.88
z=2
784.38
z=2
784.88
z=2
785.38
z=2
m/z

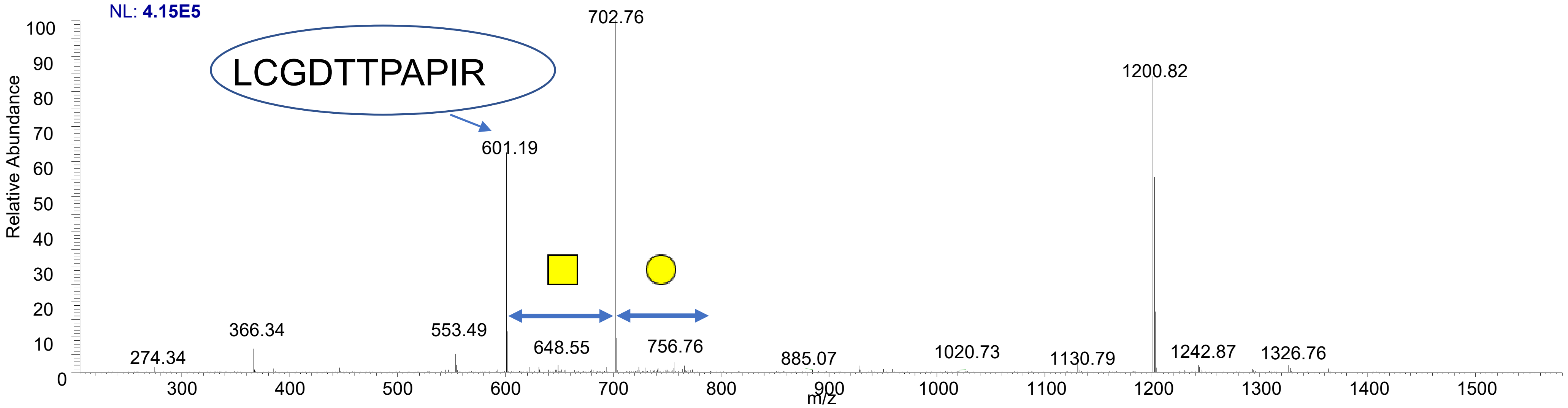

NL: 4.15E5
LCGDTTPAPIR
702.76
1200.82
601.19
274.34
366.34
553.49
648.55
756.76
885.07
1020.73
1130.79
1242.87
1326.76
Relative Abundance
m/z

Figure S3

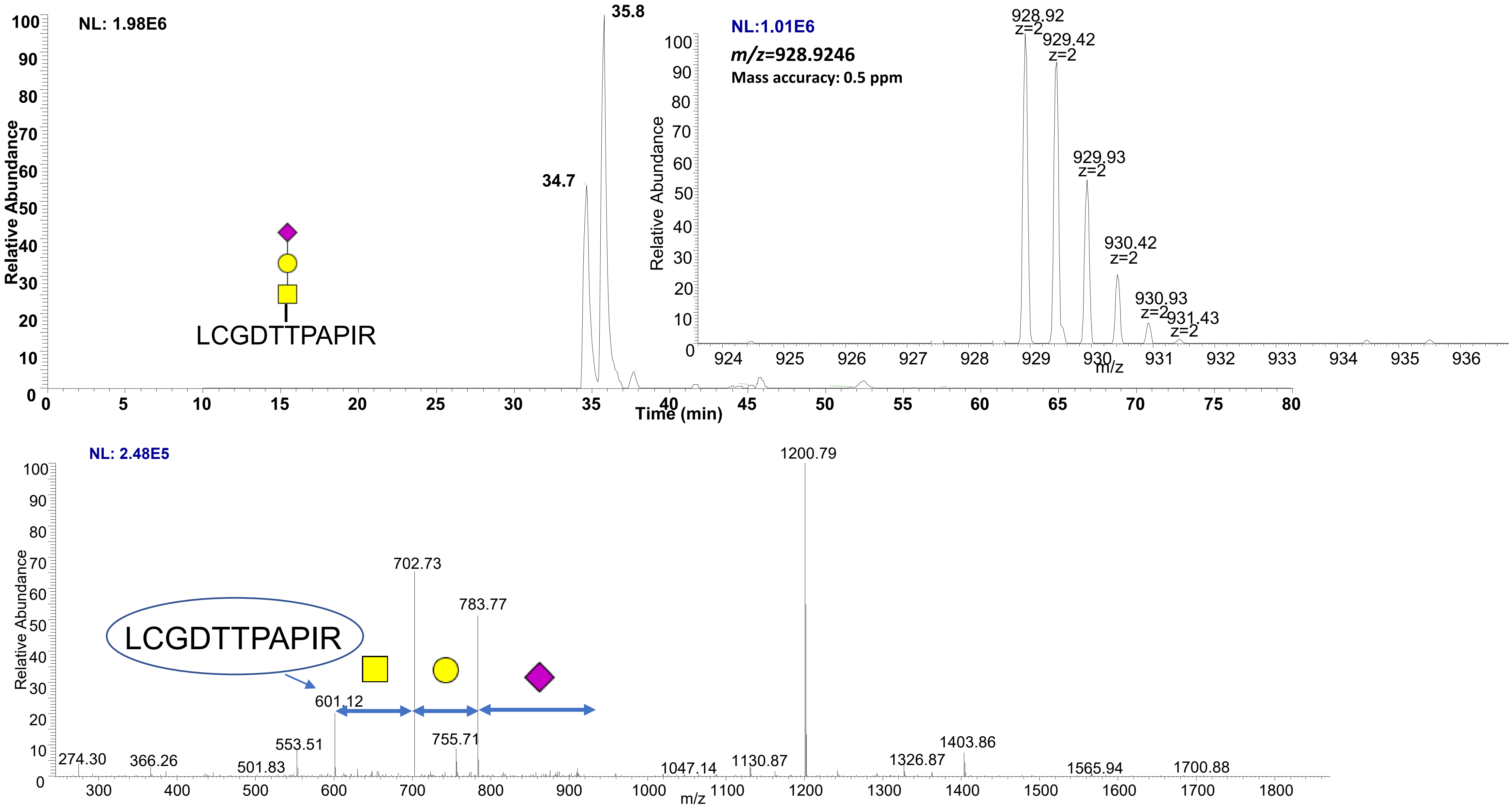
NL: 1.98E6
35.8
34.7
LCGDTTPAPIR
Relative Abundance
Time (min)
NL:1.01E6
m/z=928.9246
Mass accuracy: 0.5 ppm
928.92 z=2
929.42 z=2
929.93 z=2
930.42 z=2
930.93 z=2
931.43 z=2
m/z
NL: 2.48E5
1200.79
702.73
783.77
LCGDTTPAPIR
601.12
755.71
553.51
274.30
366.26
501.83
1047.14
1130.87
1326.87
1403.86
1565.94
1700.88
m/z

Figure S3
