# Supplementary material for: Glycosylation of a key cubilin Asn residue results in reduced binding to albumin
Source: J Biol Chem. 2022 Aug 13;298(10):102371. doi: 10.1016/j.jbc.2022.102371 (PMC9485058; doi:10.1016/j.jbc.2022.102371)
Supplement: Supplemental Figure S4 [file mmc10.pdf]

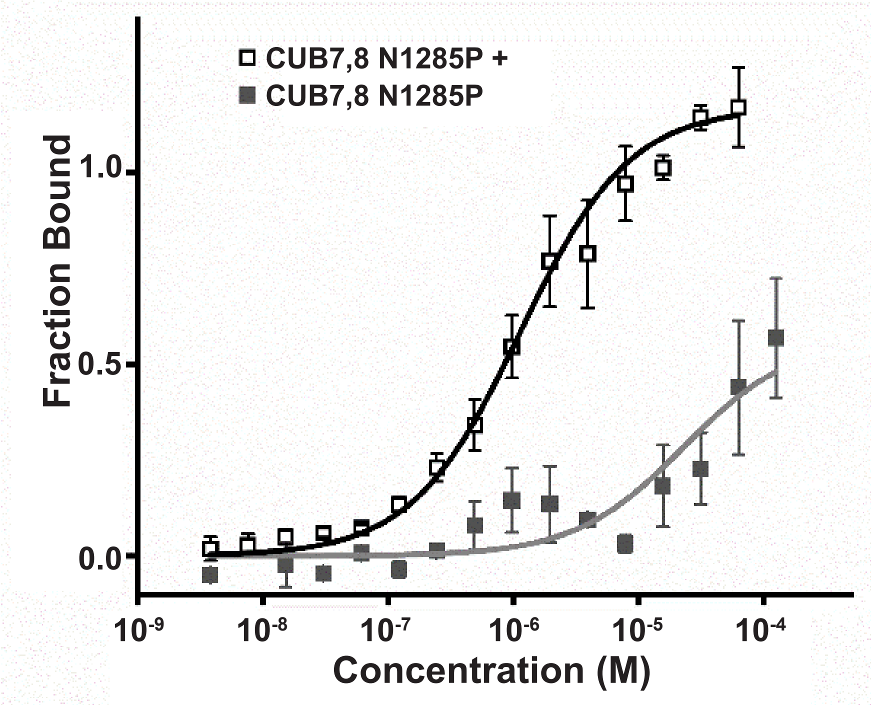

Figure S4- Binding of CUB7,9 N1285P to albumin before, solid square and after, open square, PNGaseF treatment, **+**.
