# Supplementary material for: Glycosylation of a key cubilin Asn residue results in reduced binding to albumin
Source: J Biol Chem. 2022 Aug 13;298(10):102371. doi: 10.1016/j.jbc.2022.102371 (PMC9485058; doi:10.1016/j.jbc.2022.102371)
Supplement: Supplemental Figure S5 [file mmc11.pdf]

Figure S5

INKECCHGDLLE(3)-KGFK(1)

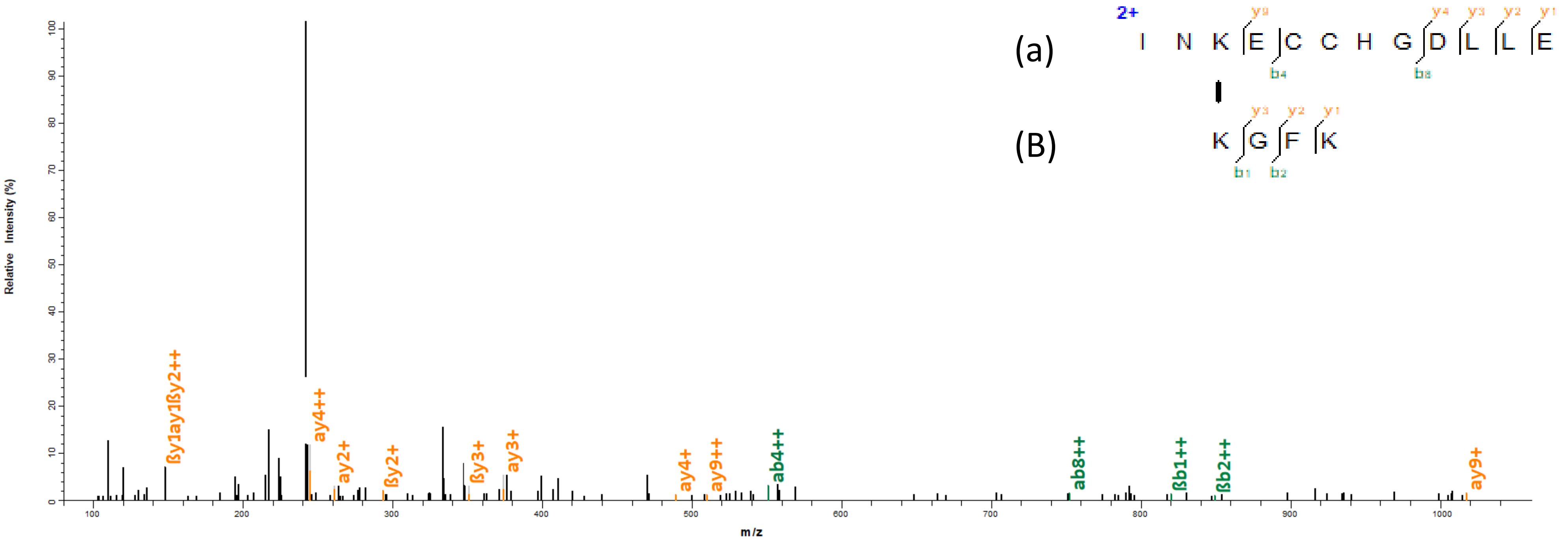

Cross-linked peptides were searched using pLink software. Spectra were generated using the pLable software from Institute of Computing Technology, Chinese Academy of Sciences, Beijing, China. Fragments are b and y ions as labeled on the right corner. Letter a and B before b, y fragments denotes the peptide (a), and (B) of cross-linked peptide.

NVVIVNKTSGILE(7)-PKNLVK(2)

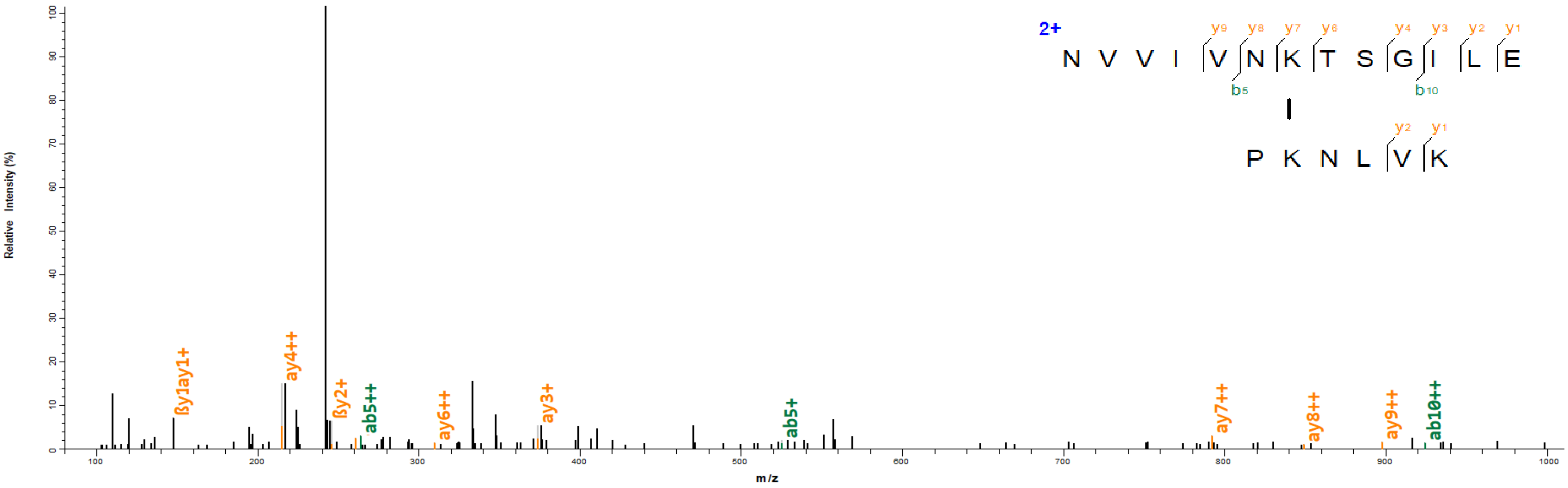

Figure S5

SINYPNPYDKNQR(10)-FAKTCVADENAE(3)

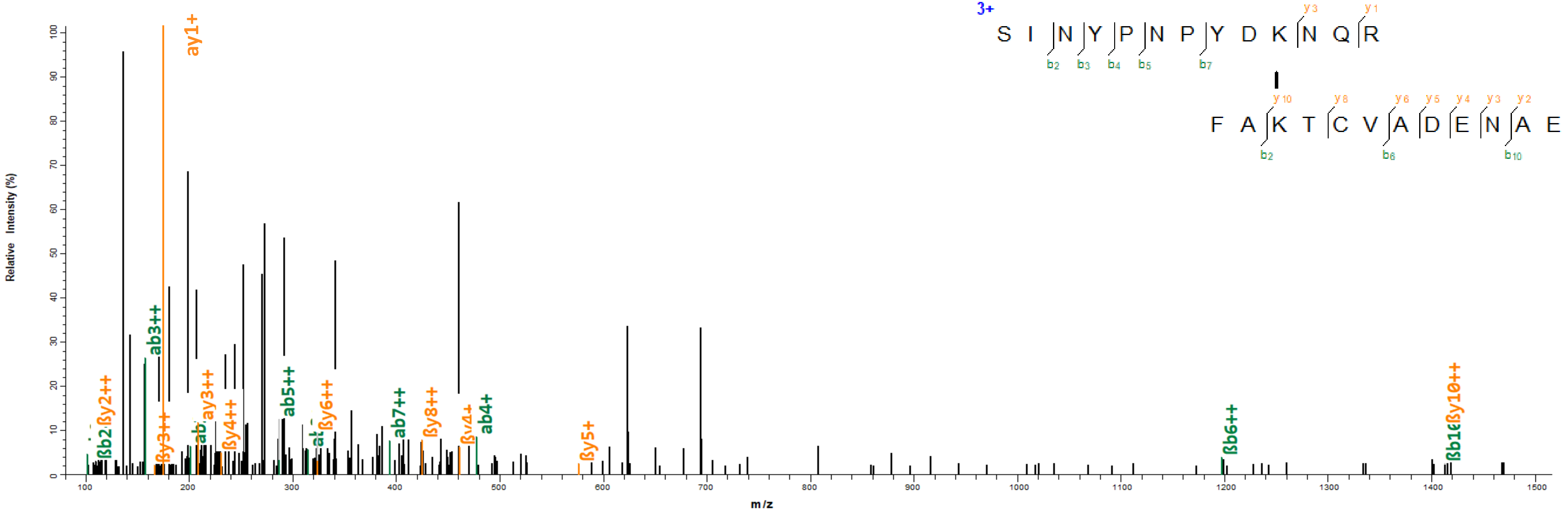

Figure S5

GFKMQWFTHGHHHH(3)-LDAVKE(5)

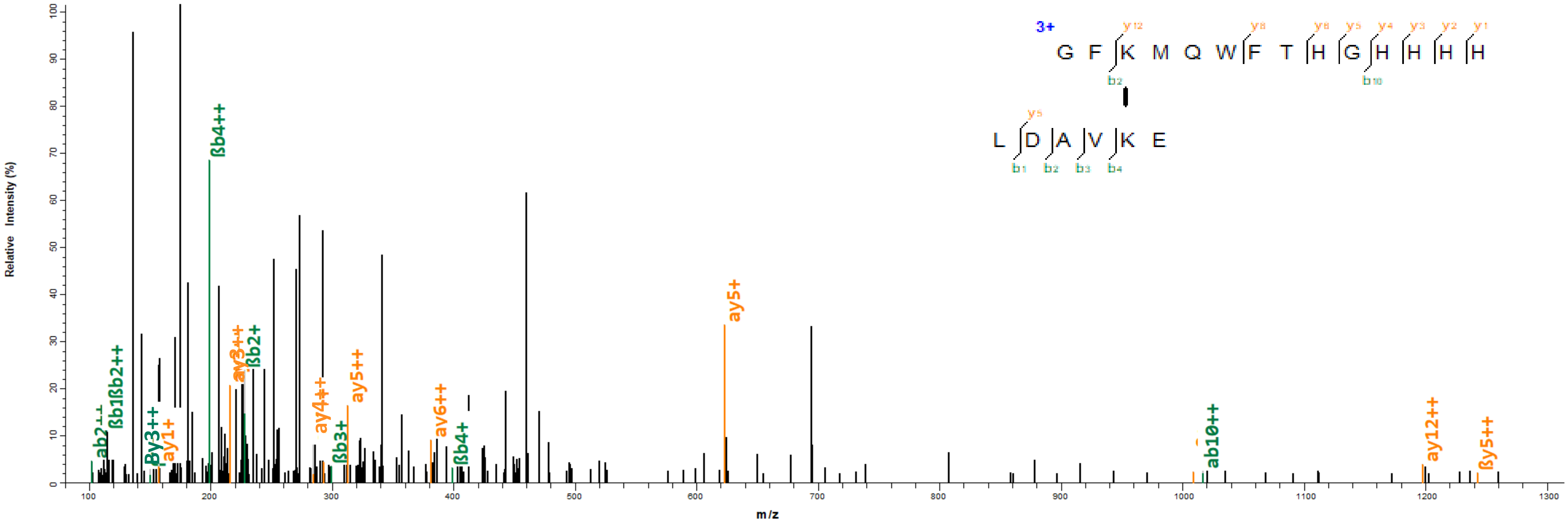

Figure S5

GINSGEKGFK(7)-DQLKTVMGD(4)

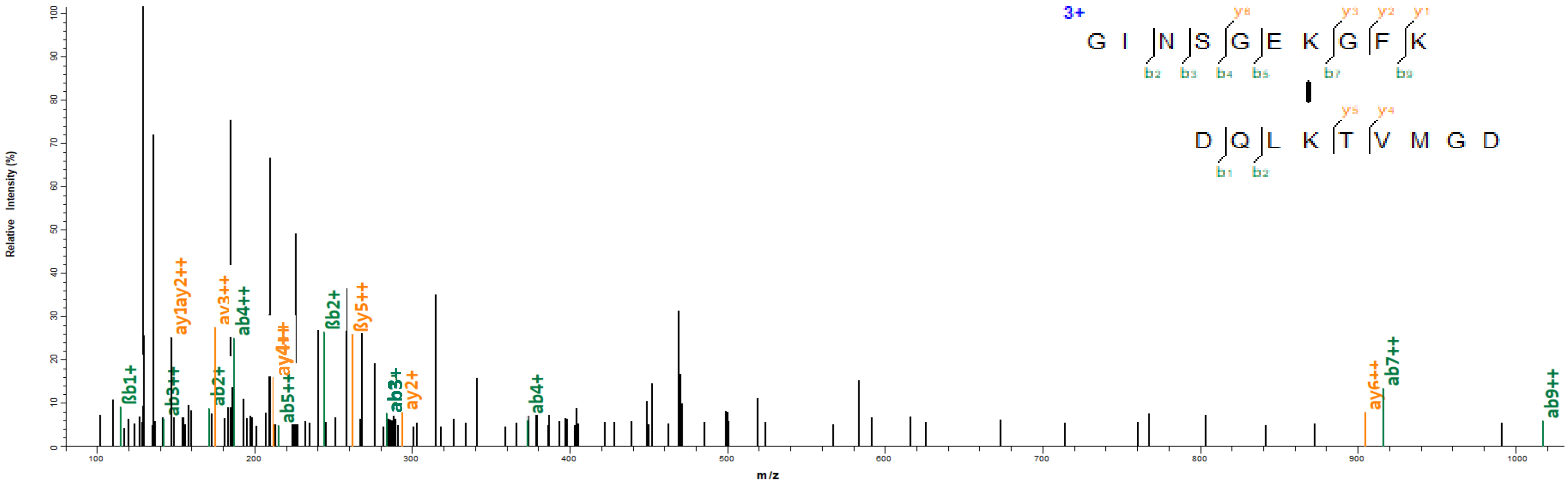

Figure S5
