# Supplementary material for: Glycosylation of a key cubilin Asn residue results in reduced binding to albumin
Source: J Biol Chem. 2022 Aug 13;298(10):102371. doi: 10.1016/j.jbc.2022.102371 (PMC9485058; doi:10.1016/j.jbc.2022.102371)
Supplement: Supplemental Figure S6 [file mmc12.pdf]

Figure S6, CUB78 binds to albumin domain III (DIII) and DIII mutant

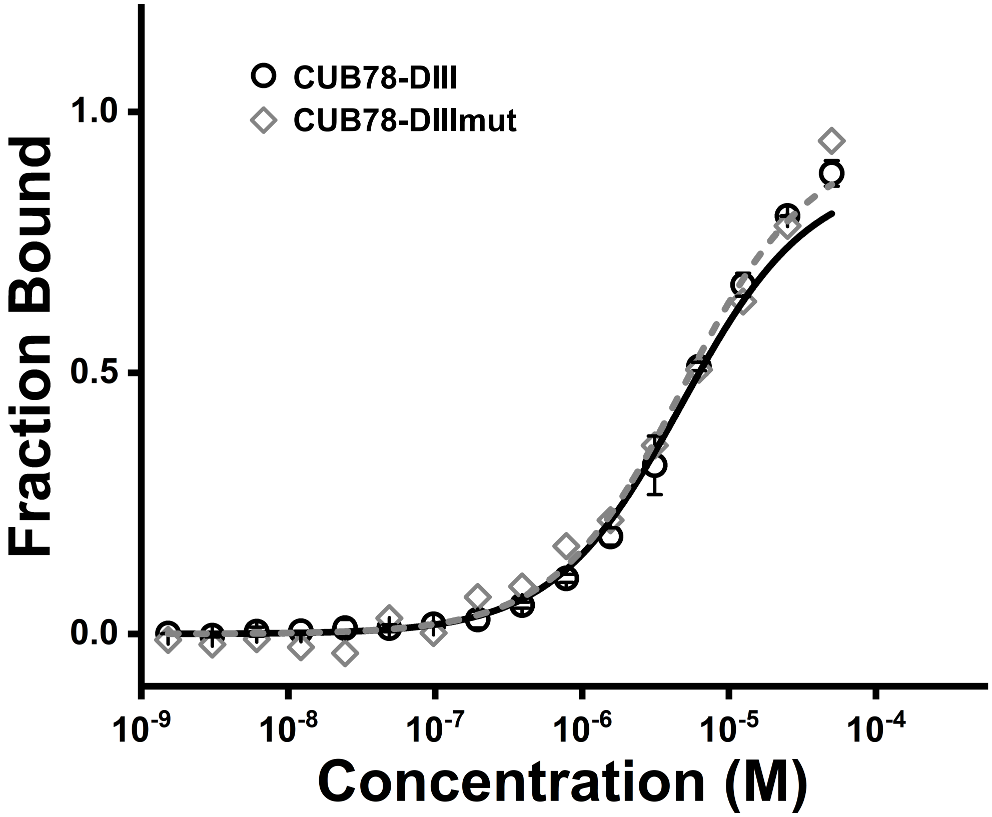

Albumin Domain III and an DIIImut that does not bind FcRn (37) was used to determine binding affinity.
