# Supplementary material for: Glycosylation of a key cubilin Asn residue results in reduced binding to albumin
Source: J Biol Chem. 2022 Aug 13;298(10):102371. doi: 10.1016/j.jbc.2022.102371 (PMC9485058; doi:10.1016/j.jbc.2022.102371)
Supplement: Supplemental Figure S7 [file mmc13.pdf]

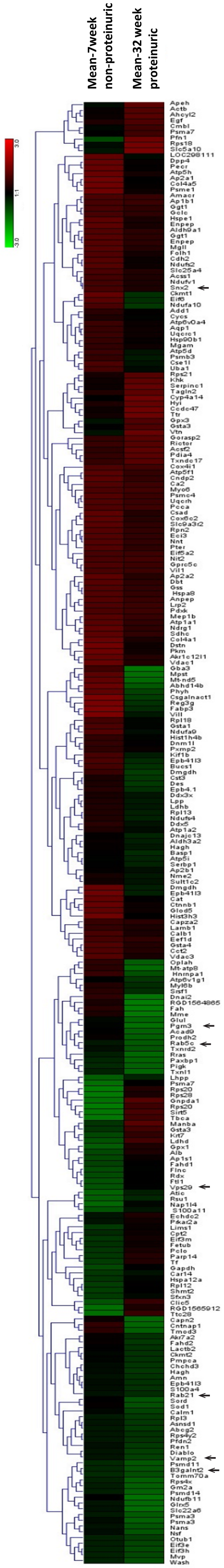

Figure S7, Heat Map of mean values for the significant ($p<0.05$) kidney cortex proteins that differed between the nonproteinuric and proteinuric MWF male rats.

The software we use to create heatmaps is Genesis (v1.8.1)
https://genome.tugraz.at/genesisclient/genesisclient_description.shtml
Up-regulated abundance in shown in red color, whereas the down-regulated are depicted in green color. A Hierarchical Clustering was calculated which clusters the proteomics data according to their trend of regulation.
The up-regulated proteins of the replicates within the same group will be clustered, and the same for the down-regulated ones.
The arrows identify the proteins mentioned in the paper B3galnt2, Rab5c, Rab21, Vamp2, Snx2, Vps29 and Pgm3.
